# Demonstrating a systems approach for integrating disparate data streams to inform decisions on children’s environmental health

**DOI:** 10.1186/s12889-022-12682-3

**Published:** 2022-02-15

**Authors:** Elaine A Cohen Hubal, Nicole M DeLuca, Ashley Mullikin, Rachel Slover, John C Little, David M Reif

**Affiliations:** 1 Center for Public Health and Environmental Assessment, US EPA, Research Triangle Park, NC USA; 2 Department of Civil and Environmental Engineering, Virginia Tech, Blacksburg, VA USA; 3 Department of Biological Sciences, North Carolina State University, Raleigh, NC USA

**Keywords:** Chemical exposure, ANOVA, ToxPi, Children’s health, Environmental health, Systems approach, Multivariate analysis, Data-driven

## Abstract

**Background:**

The use of systems science methodologies to understand complex environmental and human health relationships is increasing. Requirements for advanced datasets, models, and expertise limit current application of these approaches by many environmental and public health practitioners.

**Methods:**

A conceptual system-of-systems model was applied for children in North Carolina counties that includes example indicators of children’s physical environment (home age, Brownfield sites, Superfund sites), social environment (caregiver’s income, education, insurance), and health (low birthweight, asthma, blood lead levels). The web-based Toxicological Prioritization Index (ToxPi) tool was used to normalize the data, rank the resulting vulnerability index, and visualize impacts from each indicator in a county. Hierarchical clustering was used to sort the 100 North Carolina counties into groups based on similar ToxPi model results. The ToxPi charts for each county were also superimposed over a map of percentage county population under age 5 to visualize spatial distribution of vulnerability clusters across the state.

**Results:**

Data driven clustering for this systems model suggests 5 groups of counties. One group includes 6 counties with the highest vulnerability scores showing strong influences from all three categories of indicators (social environment, physical environment, and health). A second group contains 15 counties with high vulnerability scores driven by strong influences from home age in the physical environment and poverty in the social environment. A third group is driven by data on Superfund sites in the physical environment.

**Conclusions:**

This analysis demonstrated how systems science principles can be used to synthesize holistic insights for decision making using publicly available data and computational tools, focusing on a children’s environmental health example. Where more traditional reductionist approaches can elucidate individual relationships between environmental variables and health, the study of collective, system-wide interactions can enable insights into the factors that contribute to regional vulnerabilities and interventions that better address complex real-world conditions.

**Supplementary Information:**

The online version contains supplementary material available at 10.1186/s12889-022-12682-3.

## Background

Although the research community is recognizing that environmental health is influenced by a complex system of interdependent physical and social systems, application of systems science to support policy decisions remains limited [1]. Lang and Rayner [2] argue that big public health challenges require complex ecological thinking. They advocate for the need to address inherent complexity across four dimensions of existence; the material, biological, cultural, and social. Little,  et al. [3] propose a modeling framework that would enable integration of complex information across multiple social, economic and environmental systems. Rosenthal, et al. [4] argue that a systems science approach offers powerful, underutilized tools to develop guidance for intervention design and implementation to address global environmental health priorities. These researchers identify systems dynamic modeling methodologies and chart a path for applying these tools to address complex environmental health problems. The goal of these proposals is to extend the scope of considerations that support robust policy decisions and actions.

Though important limits remain to fully realize the potential for using system science to enable environmental health policy [5–7], available data and modeling infrastructure is rapidly advancing. Currently, significant investments are focused on providing researchers access to biomedical data required to translate from basic to public health science [8, 9]. Significant technological and methodological advancements have been made in the use of complex systems modelling in the health sector [10]. Environmental health researchers are turning to these data and methods to infer actionable insights that cannot be obtained through more reductionists and static analytical approaches.

An immediate challenge is to demonstrate that accessible data and tools can provide systems-based insights and be used today by environmental and public health policy makers and practitioners to inform actions. Here we demonstrate application of a general systems model for assessing children’s environmental health using publicly available data on social environment, physical environment, and health outcomes. The model is implemented using a publicly accessible tool designed to integrate disparate data streams and weight indicators for complex systems in a semiquantitative fashion to inform environmental health decisions across a range of dimensions and perspectives. Results of this approach are compared to a more reductionist approach (i.e., ANOVA) and advantages for policy makers are discussed.

## Methods

### Children’s Environmental Health (CEH) Systems Model

**Fig. 1:**
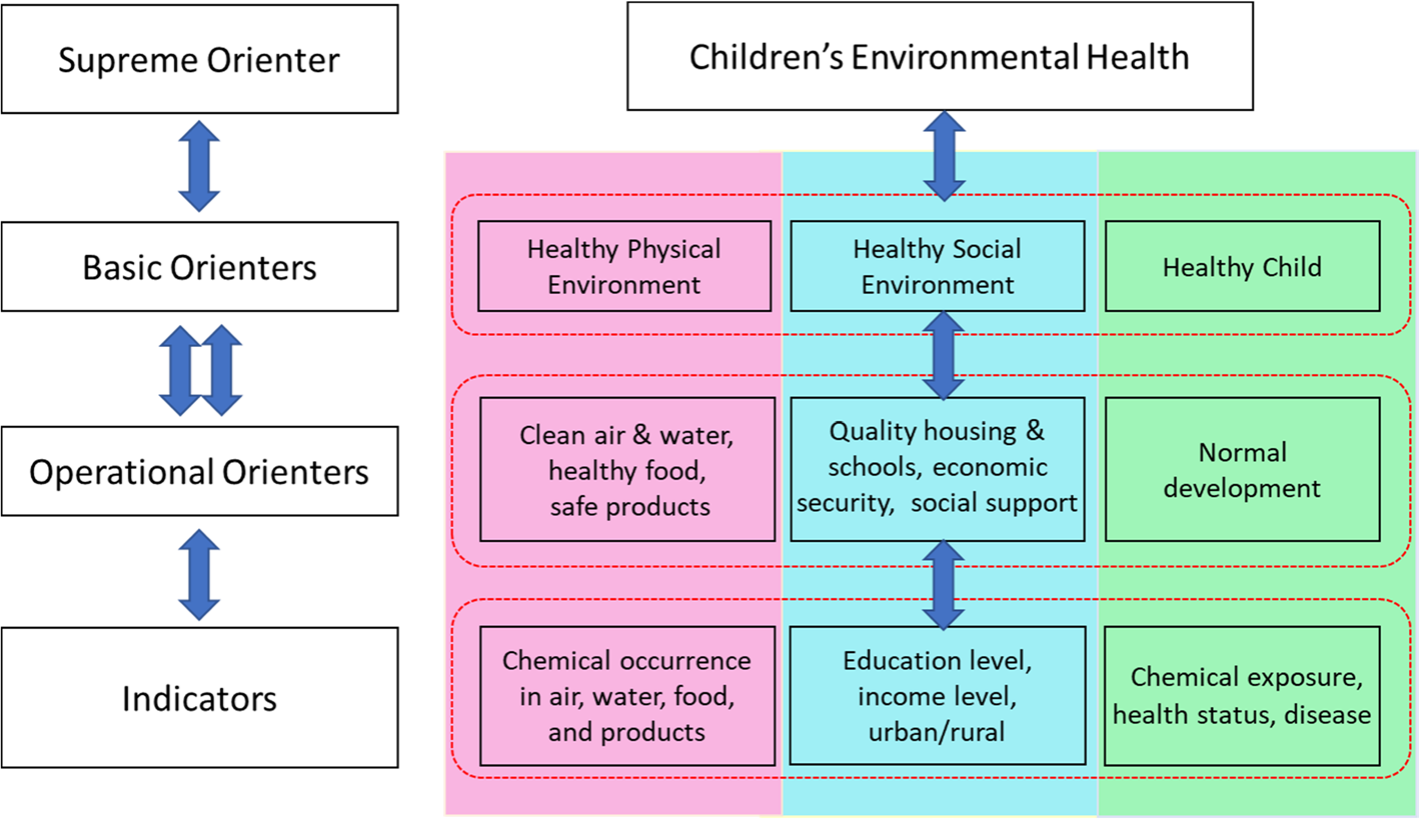
Systems thinking in children’s environmental health (Cohen Hubal et al. 2020). On the left is the systems thinking framework to which the CEH flowchart on the right corresponds. Each level breaks down across the physical environment, social environment, and child’s health to outline the necessary components of a healthy environment for child development and the relative indicators of environmental health for each of the basic orienters

A conceptual system-of-systems modeling approach for assessing children’s environmental health was previously described in Cohen Hubal, et al. [11], based on the approach described in Little, et al. [3]. Briefly, a supreme orienter, or a goal of managing a complex socioenvironmental problem, is defined. Basic orienters, or contributors to achieving the goal, are then defined to help characterize the supreme orienter. Where basic orienters can be abstract and broad, operational orienters represent a range of more specific goals and concepts encompassed by the basic orienters that can be more easily quantified. Finally, indicators, or associated quantifiable data, are identified that can be compared to the operational orienters to assess progress. The system-of-systems model used for assessing children’s environmental health in this demonstration is shown in Fig. 1.

In this model the desired goal, optimal children’s environmental health in North Carolina counties, is embodied in the following basic orienters: a healthy physical environment, a healthy social environment, and a healthy child or group of children. Operational orienters for a healthy physical environment include clean air, clean water, healthy food, and safe products. Quality of a social environment is a function of the social and economic resources available to children. The operational orienter for a healthy child is the realization of their full potential and developing physically and emotionally within a normal range. Key indicators can be identified and measured to characterize the actual state of these operational orienters such as chemical occurrence in the physical environment orienter, income level in the social environment orienter, or presence of disease in the normal developmental orienter. Indicators like these allow evaluation of the impacts of environmental health decisions and actions on the orienters and on the overall complex system governing children’s environmental health. A set of example indicators for each of the basic orienters were selected to demonstrate this approach as described below and as presented again in Fig. S1.

### Physical environment

The three indicators selected to represent chemicals in the physical environment in North Carolina counties were: (1) number of Brownfield locations, (2) number of Superfund sites, and (3) percentage of homes built before 1979. Data on Brownfield locations in each county were obtained from the North Carolina Department of Environmental Quality [12] and were manually recorded in an Excel file. Where counties reported duplicate Brownfield locations with the same name and address, only one location was recorded. Data on the number and locations of Superfund sites were obtained from the Environmental Protection Agency’s National Priorities List [13]. Data were filtered to North Carolina locations and addresses were used to manually match locations to the corresponding counties. The number of Brownfield locations and Superfund sites were summed for each county. Data on the percent of homes built before 1979 in each county were downloaded from PolicyMap [14].

### Social environment

The three indicators selected to represent the social environment in North Carolina counties were (1) percentage of residents below 18 years of age living in poverty, (2) percentage of residents under the age of 19 living without health insurance, and (3) percentage of heads of household without a high school diploma. These data were all collected from the Kids Count database through the Annie E. Casey Foundation [15]. Data on percentage of heads of household without a high school diploma were downloaded as 5-year averages from 2010 to 2014. A 5-year average was calculated across years 2011 to 2015 for percent uninsured minors in each county and percent of minors in poverty in each county.

Population size for children ages 0-4, ages 5-9, and ages 10-14 for each county were obtained from the 2019 American Community Survey through Social Explorer [16]. This database also provided a total population size for each county including both children and adults of any age. Percentage of children under age 5 in each county was calculated by dividing the population of children ages 0-4 by the total (children and adult) population.

### Health outcomes

The three indicators selected to represent children’s health in North Carolina counties were (1) percentage of low birthweight births, (2) percentage of children under age 2 with elevated blood lead levels (≥ 5 µg), and (3) percentage of children under age 15 who were reported as asthma-related hospital discharges. These data were all obtained from the Kids Count database through the Annie E. Casey Foundation [15]. The blood lead data were downloaded as 5-year averages over years 2014 to 2018. Asthma discharge data for each county were downloaded as 5-year averages over years 2010 to 2014 and were divided by the total population of children (under age 19) in each county. Percentage of low birthweights in each county was downloaded for years 2011 to 2015 and calculated as a 5-year average.

### ANOVA analysis

Analysis of variance ((ANOVA) tests are commonly used to help determine which predictor variable data best explain variability in the response data. Here we use ANOVA to investigate potential drivers of variability in children’s health outcome data from physical environment factors, social environmental factors, and other health outcomes. The normality assumption for all data described above was confirmed using QQ-plots. One-way ANOVAs were conducted for each health outcome variable against the remaining variables. The p-values were considered significant if less than 0.05. The ANOVA tests returned p-values for the social, physical, and health outcome variables in relation to one children’s health outcome, which indicates how likely the hypotheses of correlation between these different outcomes are. All calculations described in this and previous sections were performed in R (version 4.0.3).

### ToxPi Analysis

To demonstrate how integrating disparate data streams and weighing indicators for complex systems in a semiquantitative fashion can inform environmental health decisions, the ToxPi framework was used as a comparative analysis to extend univariate ANOVA results. All physical environment, social environment, and health data outlined above were loaded into the ToxPi Graphical User Interface (GUI) tool [17] and analyzed within the program’s unique statistical framework [18]. This framework provided a dimensionless index score (ToxPi score) for each county in North Carolina that is the cumulative representation of vulnerability based on the collective values of the respective vulnerability metrics. The ToxPi chart for each county is presented as a unit circle separated into different colored slices that represent each data metric. For each slice, distance from the origin is proportional to the normalized value of the component data points comprising that slice, while the width indicates the relative weight of that slice in the overall ToxPi calculation [18]. In this study, all data metrics were weighted equally, as visualized by equivalent radial widths for each slice.

Knowledge-driven exploration of the individual metrics was facilitated through ToxPi’s ranking and visualization of each of the counties. The ToxPi output was imported into R, where K-means cluster plots (Fig. S3) were used to determine the optimal number of groups for the North Carolina counties. A hierarchical clustering analysis within ToxPi (Fig. S4) was used to confirm the decision to use 5 groups of counties and perform the grouping. The grouped county data were exported from ToxPi to be mapped via the ToxPi*GIS web application (https://toxpi.org/gis/webapp/), using the latitude and longitude coordinates for the center of each North Carolina county. To visualize the relationship between the vulnerability indicators (physical environment, social environment, and health outcomes) and the distribution of children in North Carolina, the ToxPi results were plotted over a base map showing the percent of population under age 5 in each county. The base map was created in ArcMap Online, then transferred into ToxPi*GIS. The hierarchical clusters from the ToxPi analysis were then overlayed on the base map to visualize trends in the social environment, physical environment, and health outcomes across the state.

## Results

### ANOVA

Table 1 shows the results of the ANOVA analysis for three health outcomes used as dependent variables – low birthweight rates, asthma hospital discharges, and blood lead levels. The results describe relationships between each of these health outcomes and the remaining physical environment, social environment, and health outcome variables for all counties in North Carolina. Two social environment factors, percent in poverty under age 19 and percent uninsured under age 19, as well as the percent of children’s asthma hospital discharges were statistically significant in describing low birthweight rates. The same two social environment factors, percent in poverty under age 19 and percent uninsured under age 19, as well as the number of Brownfield sites and the percent of low birthweights were statistically significant in describing children’s asthma hospital discharge rates. One social environment factor, percent in poverty under age 19, and one physical environment factor, percent of homes built before 1979, are statistically significant in describing children’s elevated blood lead level rates. Neither of the other two health outcomes were statistically significant in the elevated blood lead model results.

**Table 1:** Pairwise ANOVA Results for Social Environment, Physical Environment, and Health Outcome Variables (bold indicates statistical significance as p <0.05)

Indicators	Low Birthweight Rates	Asthma Hospital Discharges_*a*_	Blood Lead Levels_*b*_
Poverty_*c*_	**2.00E-16**	**3**.32**E-07**	**1.01E-05**
Uninsured_*d*_	**2**.89**E-06**	**1.3**4**E-03**	0.34
Highschool Education_*e*_	0.11	0.17	0.07
Superfund Sites	0.54	0.12	0.055
Brownfield Sites	0.16	**4.0E-02**	0.54
Homes Built Before 1979	0.82	0.17	**7.0E-03**
Low Birthweight Rates	-	**1.1E-02**	0.52
Asthma Hospital Discharges	**1.2E-02**	-	0.97
Blood Lead Levels	0.97	0.53	**-**

^a^ Children under age 15 released from the hospital due to asthma

^b^ Children under age two with elevated blood lead levels (≥ 5 µg)

^c^ Children under age 19 living below the poverty line

^d^ Children under age 19 living without insurance

^e^ Head of household without a high school education

### Systems approach

A dimensionless index score was calculated in ToxPi for each county in North Carolina. This ToxPi score is the cumulative representation of optimal children’s health in the systems model or vulnerability based on the physical environment, social environment, and health outcome metrics used in this study as presented in Fig. S1. A vulnerability ranking of all counties is determined based on these scores, where a higher ranking indicates greater vulnerability. The ToxPi ranking of the 100 North Carolina Counties is presented in Fig. S2. This ranking may be most important when considering all attributes equally as a measure of total vulnerability. Tyrrell County, Robeson County, and Scotland County are ranked highest (highest vulnerability), while Orange County, Currituck County, and Union County are ranked lowest (lowest vulnerability).

**Fig. 2:**
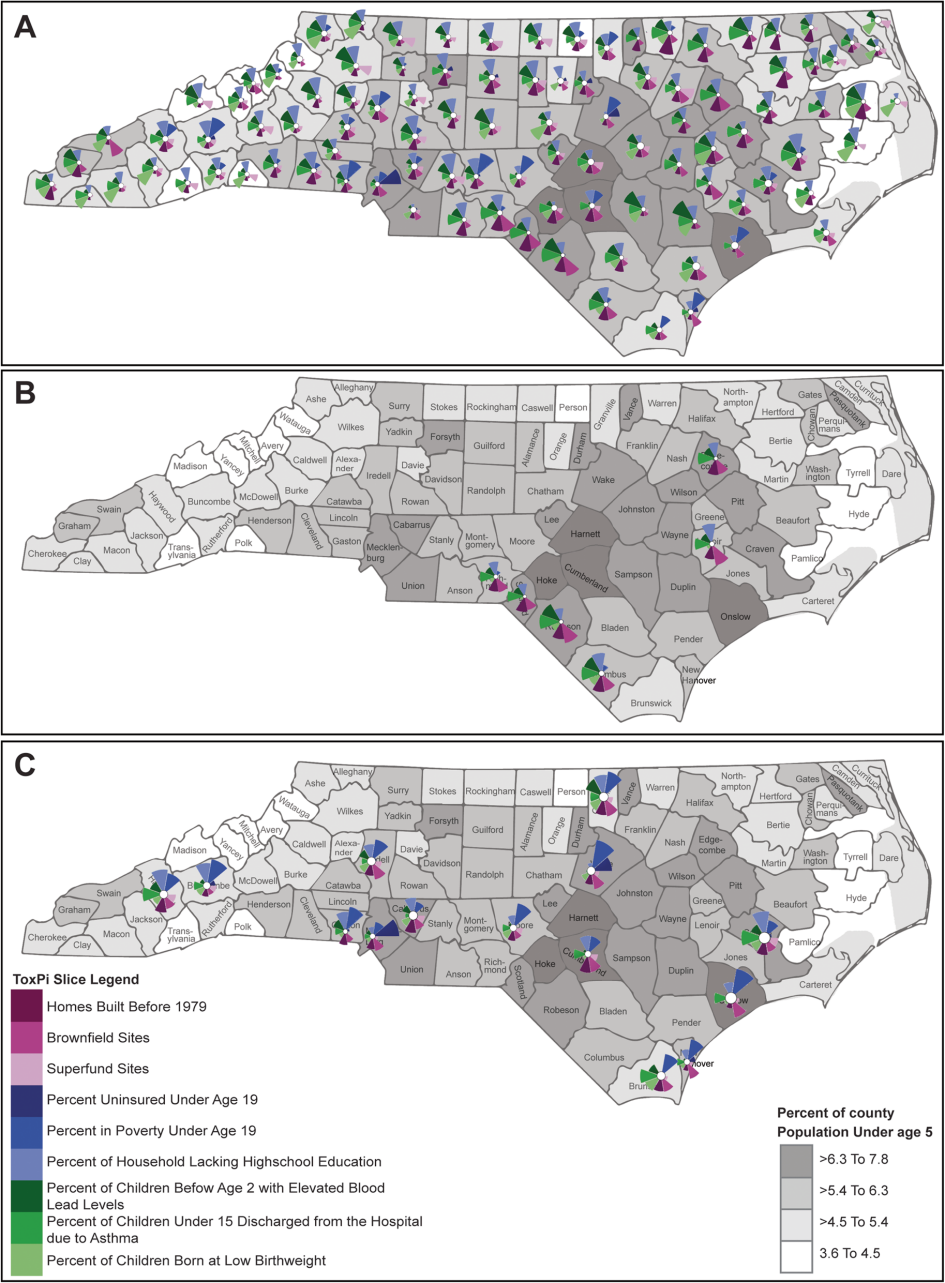
ToxPi charts overlaid on map of North Carolina Counties. **A** All Counties, **B** Group 1 results of K-means cluster analysis, **C** Group 3 results of K-means cluster analysis

ToxPi charts are produced for each county as a unit circle, on which the size (distance from origin) of the colored pie slices in the charts indicate which metrics are driving vulnerability scores (Fig. 2). The highest ranked county, Tyrrell County, is largely influenced by the percent of homes built before 1979, all three of the social environment metrics, and the percent of babies born at low birthweights (Fig. S2). The lowest ranking county, Union County, is not strongly influenced by any of the metrics used in the study (Fig. S2).

Results of the K-means clustering analysis to determine the optimal number of groups of counties with similar vulnerability characteristics is shown in Fig. S3. Along with visual investigation of similar characteristics between county charts in a hierarchical clustering analysis in ToxPi (Fig. S4), the K-means clustering analysis suggested that 5 groups of counties optimally describe this dataset (minimize within-cluster variation). A list of counties that were assigned to each of the 5 groups based on ToxPi’s hierarchical clustering analysis is shown in Table S1.

Group 1 contains 6 counties with some of the highest ToxPi vulnerability scores, all ranking 89 or greater out of the 100 North Carolina counties. These counties are located mainly along the central southern border of North Carolina except for Edgecombe and Lenoir counties that are northeast of the others, and their ToxPi charts show strong influences from all three categories of operational orienters (Fig. S5). In the social environment, these counties are influenced by percent of household heads without a high school diploma and percent of children living in poverty. The physical environment contributes to their vulnerability from percent of homes built before 1979, and health outcomes influence the ToxPi scores from percent of babies born at low birthweights and percent of children discharged from the hospital from asthma. The percent of the population that are children less than age five in these counties ranges from 5.4 to 7.8% (Fig. S5).

Group 2 contains 15 counties that have higher ToxPi vulnerability scores, with all counties ranking 67 or greater out of 100. These counties are located mainly in the northeast section of North Carolina except for Bladen and Anson counties in the southern part of the state and Graham County in the western part of the state (Fig. S6). The ToxPi charts in this group are generally characterized by strong influences from percent of homes built before 1979 in the physical environment and percent of children living in poverty in the social environment. While other influences vary between counties, many have contributions to vulnerability from percent of household heads without a high school diploma, percent of babies born at a low birthweight, and percent of children with elevated blood lead levels. The percent of the population that are children in these counties is generally below 6.3%, except for Graham and Vance counties where children under age five range from 6.3 to 7.8% of the population (Fig. S6).

The third group contains 14 counties that span a wide range of ToxPi rankings and locations across the state (Fig. S7). The ToxPi charts in this group are primarily characterized by their physical environment impact from Superfund sites, as well as a strong influence from Brownfield sites in Mecklenburg County. Of the three health indicators, percent of children discharged from the hospital from asthma appears to have more influence on vulnerability in many of these counties. The percent of children under age five in the total population of each county ranges from 4.5% up to 9.9% in Onslow and Cumberland counties (Fig. S7).

The fourth group contains 20 counties that have a lower range of ToxPi rankings spanning from 1 to 42 out of 100. Like the third group, these counties are located across the state but also include coastal counties in the eastern part of the state (Fig. S8). While small in magnitude, vulnerability in many of these counties has stronger influences from social environment indicators or percent of homes built before 1979 than the other data metrics. The percent of children in the total population of each county ranges from 3.6% up to 9.9% in Hoke and Harnett counties (Fig. S8).

Group 5 contains the remaining 45 North Carolina counties with mid-range ToxPi rankings from 17 to 84 of out 100 that span the state (Fig. S9). The ToxPi vulnerability in this group mainly has contributions from percent of homes built before 1979 and social environment indicators like percent of household heads without a high school diploma and percent of children living without health insurance. Percent of children discharged from the hospital with asthma is a strong influence on vulnerability in Swain County in the western part of the state. The percent of children under age five in these counties ranges from 3.6 to 7.8% (Fig. S9).

## Discussion

### Comparison of ANOVA and ToxPi Analyses

The associations between each of the health indicators and the social and physical environment indicators on the state-level from the ANOVA analyses (Table 1) were compared to observations from the ToxPi charts for each cluster of counties. At the state-level using ANOVA, the percent of low birthweights health indicator was statistically significant in relation to the percent of children in poverty, percent of children without health insurance, and percent of children’s asthma discharges from the hospital. At the county-level using ToxPi, a cluster of counties (Group 1, Fig. S5) had associations between vulnerability from low birthweights and percent of household heads without a high school diploma, percent of homes built before 1979, percent of children in poverty, and percent of children discharged from the hospital from asthma. ToxPi analyses showed that another cluster of counties (Group 2, Fig. S6) had associations between vulnerability from low birthweights and percent of homes built before 1979, percent of children living in poverty, and percent of household heads without a high school diploma. Unlike the ANOVA analyses, ToxPi results for these two groups of counties did not show associations between vulnerability from percent of low birthweights and percent of children without insurance. Only Group 1 showed the association between low birthweights and asthma discharges that were found in the ANOVA analysis.

At the state-level using ANOVA, the health indicator describing percent of children’s asthma discharges from the hospital was statistically significant in relation to percent of children in poverty, percent of children without insurance, number of Brownfield sites in each county and percent of low birthweights. At the county-level using ToxPi, a cluster of counties (Group 3, Fig. S7) also had an association between vulnerability from asthma hospital discharge rates and a physical environment indicator. However, the indicator shown to be significant in the ANOVA results (Brownfield sites) was different than the indicator in the ToxPi results (Superfund sites). The ToxPi results for this group did not show associations between asthma discharge rates and percent of children in poverty, percent of children without insurance, or percent of low birthweights. Another group of counties (Group 1, Fig. S5) had associations between vulnerability from asthma hospital discharge rates and percent of household heads without a high school diploma, percent of children living in poverty, percent of homes built before 1979, and percent of low birthweights. Unlike the ANOVA, the ToxPi results for this group did not show associations between vulnerability from asthma hospital discharge rates and percent of children without insurance or the number of Brownfield sites in the counties. A third group (Group 5, Fig. S9) in the ToxPi analysis results contained one county (Swain County) that showed the association between percent of children’s asthma hospital discharges and percent of children living without insurance that was found to be statistically significant in the ANOVA analysis results.

When investigating relationships between social environment, physical environment, and health outcome indicators and elevated blood lead levels in children, the state-level ANOVA results showed statistical significance with percent of children in poverty and percent of homes built before 1979. County-level ToxPi results showed one group (Group 2, Fig. S6) with associations between elevated blood lead levels and percent of children in poverty, percent of homes built before 1979, percent of household heads without a high school diploma, and percent of low birthweights. The associations between percent of children with elevated blood lead levels and percent of household heads without a high school diploma and percent of low birthweights in this group were not represented in ANOVA results.

### Implementing a Systems Approach

More generally, systems science enables researchers to integrate the rapidly expanding body of information on children’s environments with advancing insights on child development and health (Rosenthal, Payne-Sturges). In the systems-of-systems approach described by Little (Little et al. [3] and adapted in the CEH systems model considered here [11], progress towards achieving the goal (e.g., children’s environmental health) is assessed by comparing the operational orienters (the desired state of the complex system) to the associated indicators (the actual state of the complex system) and evaluating the extent of orienter satisfaction. This orienter-based approach provides a flexible and systematic method that can be expanded and adjusted as systems are added to consider additional interactions.

To fully implement a systems approach in this way, significant investments in data collection, computational model development, and expertise is required. The example presented here demonstrates that while researchers work to build these capabilities, environmental and public health practitioners can begin to apply a systems approach using accessible data and available tools. In this way, systems thinking can facilitate translating scientific information on key factors across multiple spatial and temporal scales to support decisions that promote and protect children’s health.

## Conclusions

In this study, a systems approach was presented to build on a traditional reductionist approach (pair-wise ANOVA) for evaluating children’s environmental health and gleaning insights on differences in regional vulnerabilities. An example set of indices drawn from publicly available data were used to characterize the three systems model orienters of physical environment, social environment, and health. While the ANOVA here enabled an understanding of important relationships on the State level, the ToxPi analysis allowed a view of factors influencing vulnerability at the county level. Because the systems model (ToxPi) includes indices representing both independent and dependent variable of the traditional model (ANOVA, Table 1) a direct comparison of these two analyses is not informative. Rather both approaches provide important information.

Where more traditional reductionist approaches can elucidate individual relationships between environmental variables and health, the study of collective, system-wide interactions can enable insights into the factors that contribute to regional vulnerabilities and interventions that better address complex real-world conditions. Visualization of both independent and dependent variables is a strength of this approach by pointing to problems, the driving set of factors, and potentially to interventions. When scaled up for large numbers of modifiable variables in each metric category (health, physical, social), the approach demonstrated here could be extremely valuable in supporting decisions and actions that consider children’s environmental health holistically.

## Supplementary Information

**Additional file 1.**

## Data Availability

The raw datasets used are available via the following repositories: North Carolina Department of Environmental Quality (Brownfield sites) https://deq.nc.gov/about/divisions/waste-management/brownfields-program, Environmental Protection Agency National Priorities List (Superfund sites) https://www.epa.gov/superfund/superfund-national-priorities-list-npl, PolicyMap (percent of homes built before 1979) https://www.policymap.com/data/our-data/, Annie E. Casey Foundation Kids Count database (all social and physical environmental factors) https://datacenter.kidscount.org, and Social Explorer 2019 American Community Survey (population) https://www.socialexplorer.com/explore-maps. The compiled dataset used for analysis is available from the corresponding author on reasonable request.

## Abbreviations

ToxPi: Toxicological Prioritization Index
ANOVA: Analysis of variance
GIS: Geographical Information Systems
CEH: Children’s environmental health
