# Supplementary material for: Demonstrating a systems approach for integrating disparate data streams to inform decisions on children’s environmental health
Source: BMC Public Health. 2022 Feb 15;22:313. doi: 10.1186/s12889-022-12682-3 (PMC8845296; doi:10.1186/s12889-022-12682-3)
Supplement: Supplementary file 1 — Additional file 1. [file 12889_2022_12682_MOESM1_ESM.docx]

**Supplemental Information**

Demonstrating a Systems Approach for Integrating Disparate Data Streams to Inform Decisions on Children’s Environmental Health

Elaine A Cohen Hubal, Nicole M DeLuca, Ashley Mullikin, Rachel Slover, John C Little, David M Reif

*Table S1. Counties Assigned to each Hierarchical Cluster Group*

| **Group 1** | **Group 2** | **Group 3** | **Group 4** | **Group 5** |
| --- | --- | --- | --- | --- |
| Columbus (91) | Anson (70) | Brunswick (21) | Camden (7) | Alamance (58) |
| Edgecombe (97) | Bertie (94) | Buncombe (51) | Carteret (10) | Alexander (24) |
| Lenoir (89) | Bladen (67) | Cabarrus (8) | Chatham (16) | Alleghany (83) |
| Robeson (99) | Chowan (82) | Cumberland (46) | Currituck (2) | Ashe (55) |
| Richmond (96) | Graham (79) | Craven (13) | Dare (4) | Avery (59) |
| Scotland (98) | Greene (90) | Gaston (85) | Davie (18) | Beaufort (72) |
|  | Halifax (95) | Granville (34) | Durham (30) | Burke (38) |
|  | Hertford (81) | Haywood (57) | Franklin (32) | Caldwell (49) |
|  | Martin (87) | Iredell (25) | Harnett (20) | Caswell (73) |
|  | Northampton (92) | Mecklenburg (65) | Henderson (15) | Catawba (26) |
|  | Tyrrell (100) | Moore (36) | Hoke (23) | Cherokee (44) |
|  | Vance (86) | New Hanover (40) | Johnston (9) | Clay (17) |
|  | Washington (93) | Onslow (5) | Lincoln (14) | Cleveland (62) |
|  | Warren (88) | Wake (22) | Orange (3) | Davidson (28) |
|  | Wilson (80) |  | Pender (11) | Duplin (76) |
|  |  |  | Pitt (12) | Forsyth (53) |
|  |  |  | Polk (42) | Gates (27) |
|  |  |  | Transylvania (53) | Guilford (29) |
|  |  |  | Union (1) | Hyde (69) |
|  |  |  | Watauga (6) | Jackson (35) |
|  |  |  |  | Jones (64) |
|  |  |  |  | Lee (63) |
|  |  |  |  | Macon (48) |
|  |  |  |  | Madison (39) |
|  |  |  |  | McDowell (37) |
|  |  |  |  | Mitchell (71) |
|  |  |  |  | Montgomery (84) |
|  |  |  |  | Nash (54) |
|  |  |  |  | Pamlico (52) |
|  |  |  |  | Pasquotank (45) |
|  |  |  |  | Perquimans (31) |
|  |  |  |  | Person (50) |
|  |  |  |  | Randolph (47) |
|  |  |  |  | Rockingham (68) |
|  |  |  |  | Rowan (61) |
|  |  |  |  | Rutherford (43) |
|  |  |  |  | Sampson (75) |
|  |  |  |  | Stanly (60) |
|  |  |  |  | Stokes (19) |
|  |  |  |  | Surry (74) |
|  |  |  |  | Swain (77) |
|  |  |  |  | Wayne (41) |
|  |  |  |  | Wilkes (66) |
|  |  |  |  | Yadkin (78) |
|  |  |  |  | Yancey (56) |


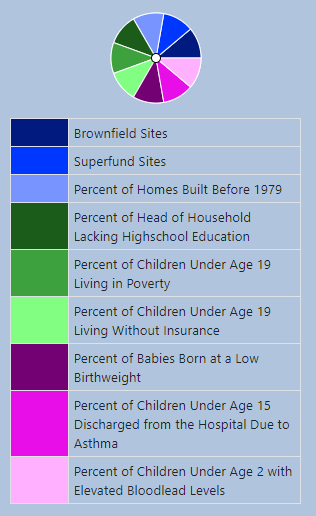


*Figure S1. ToxPi Slice Legend*

| 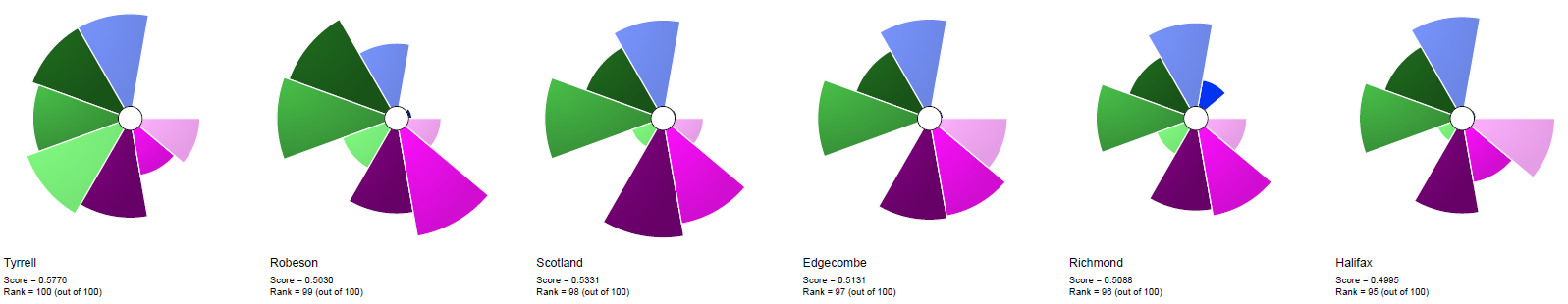  Tyrrell  Rank = 100 | 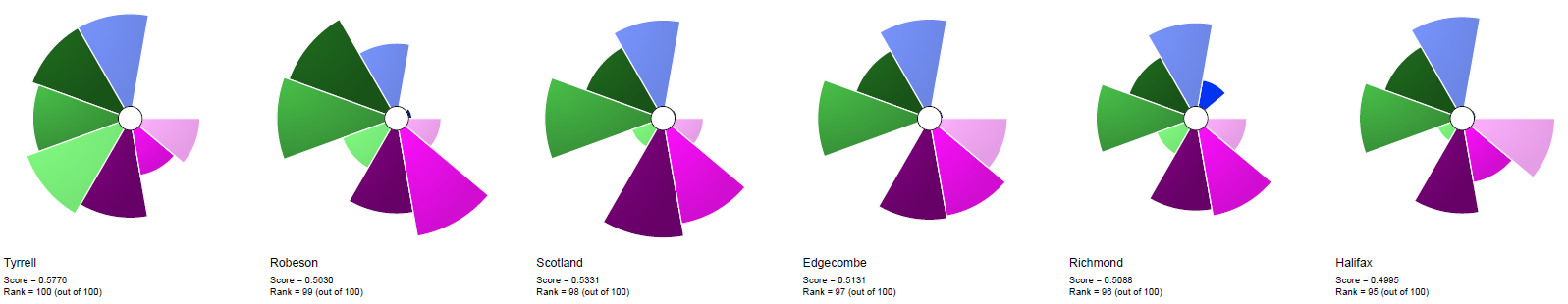  Robeson  Rank = 99 | 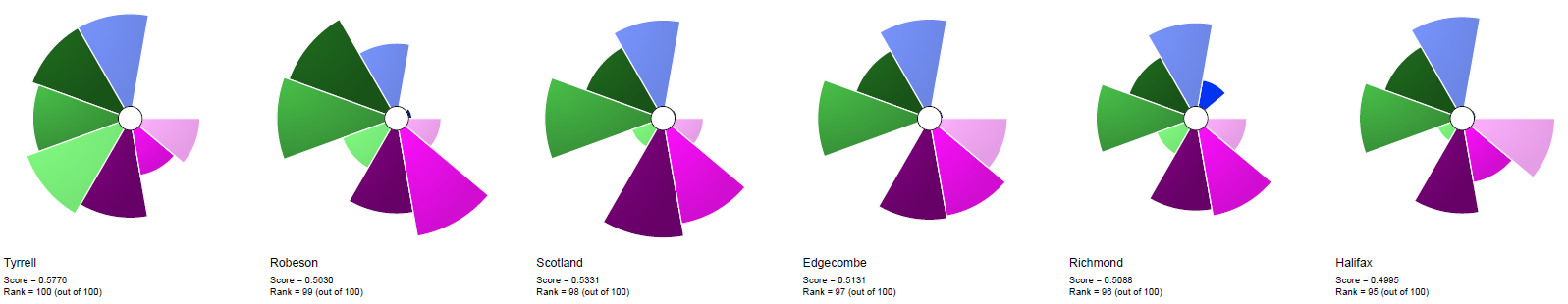  Scotland  Rank = 98 | 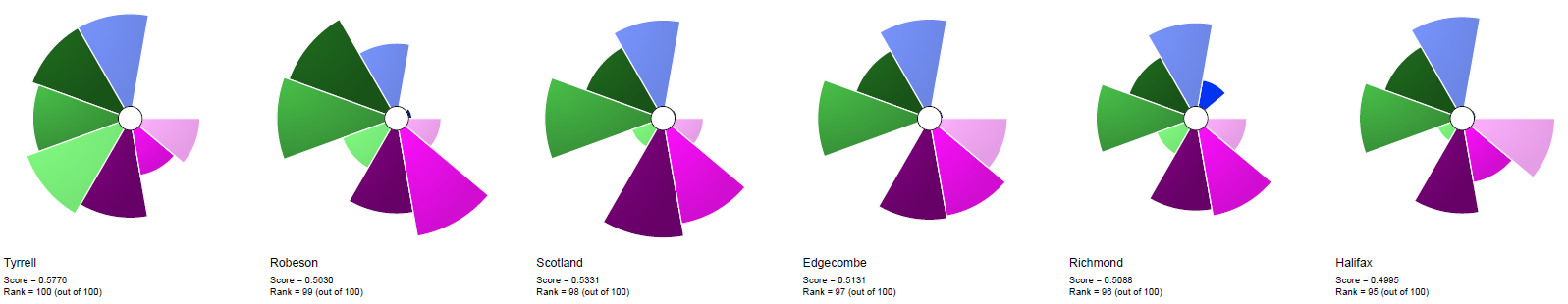  Edgecombe  Rank = 97 | 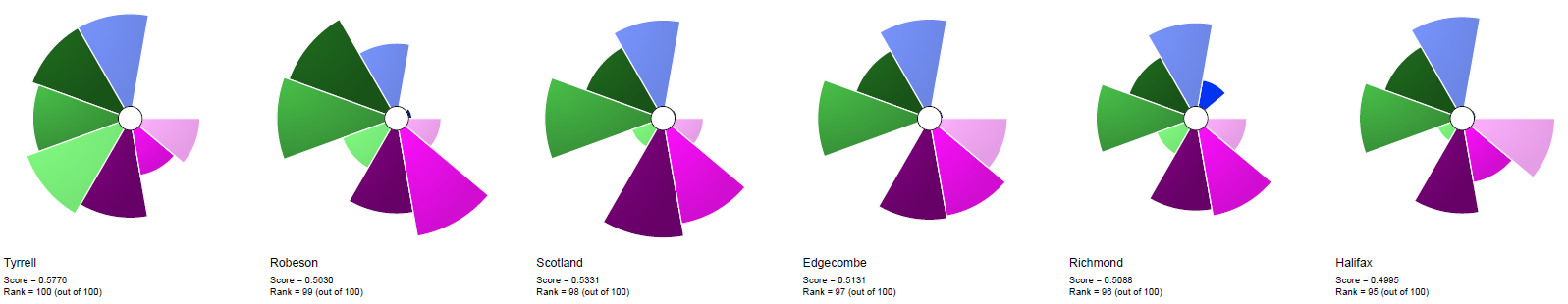  Richmond  Rank = 96 |
| --- | --- | --- | --- | --- |
| 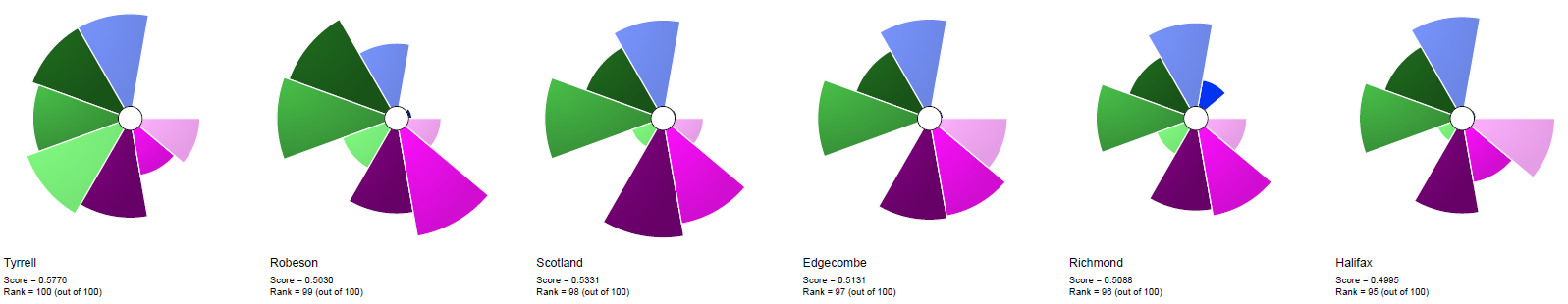  Halifax  Rank = 95 | 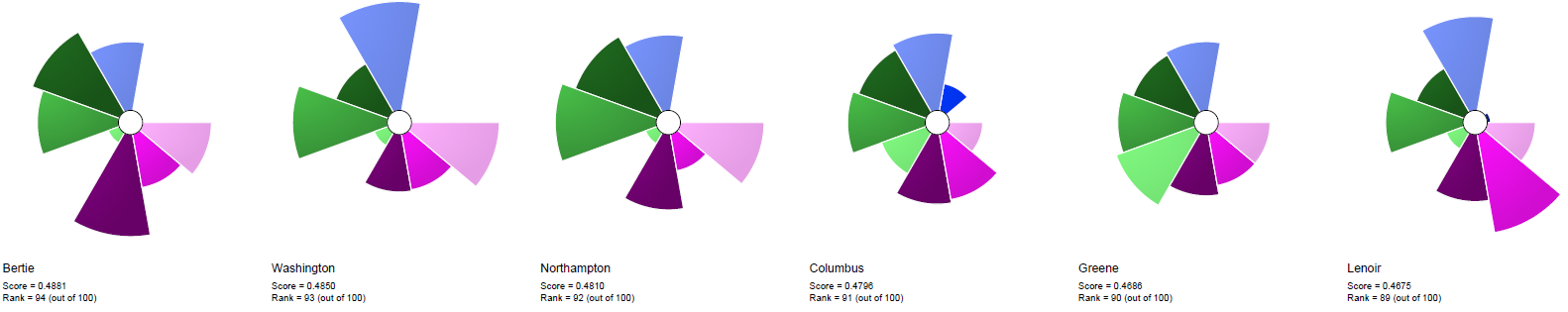  Bertie  Rank = 94 | 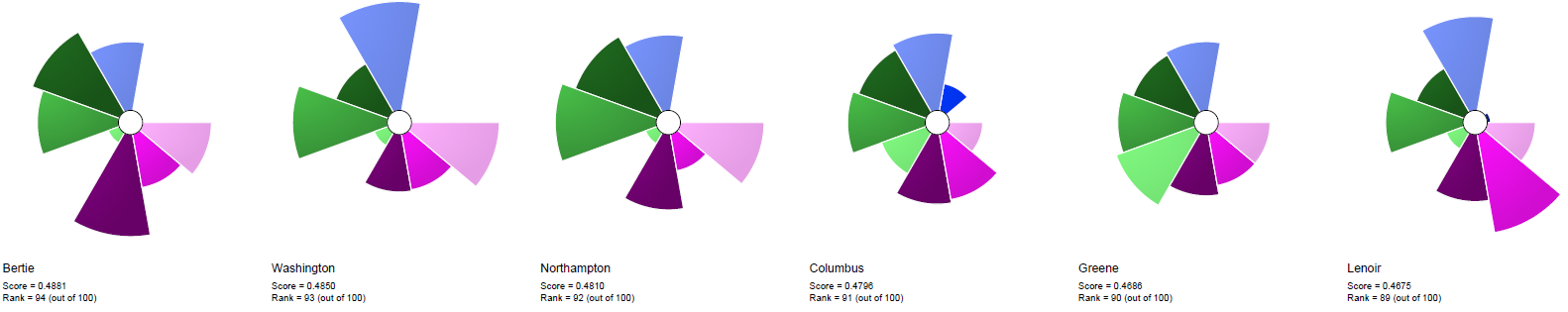  Washington  Rank = 93 | 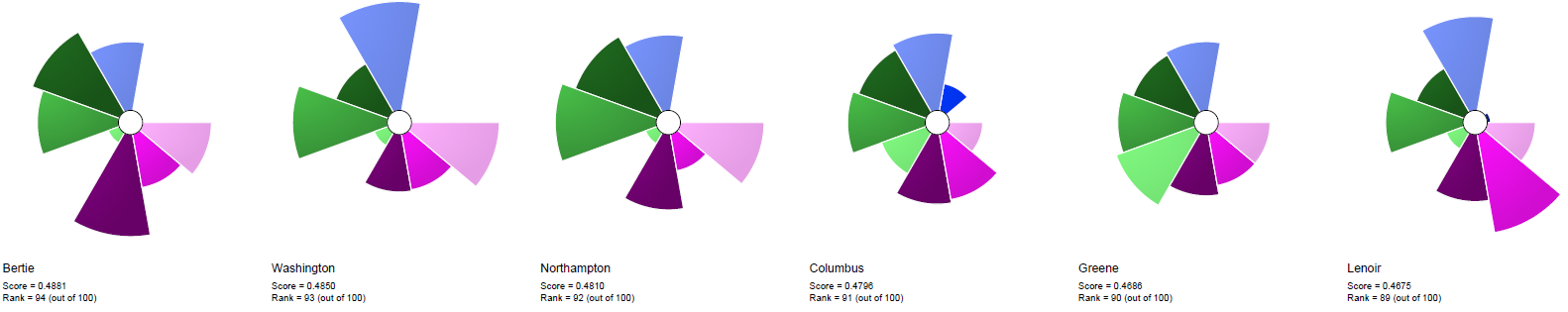  Northampton  Rank = 92 | 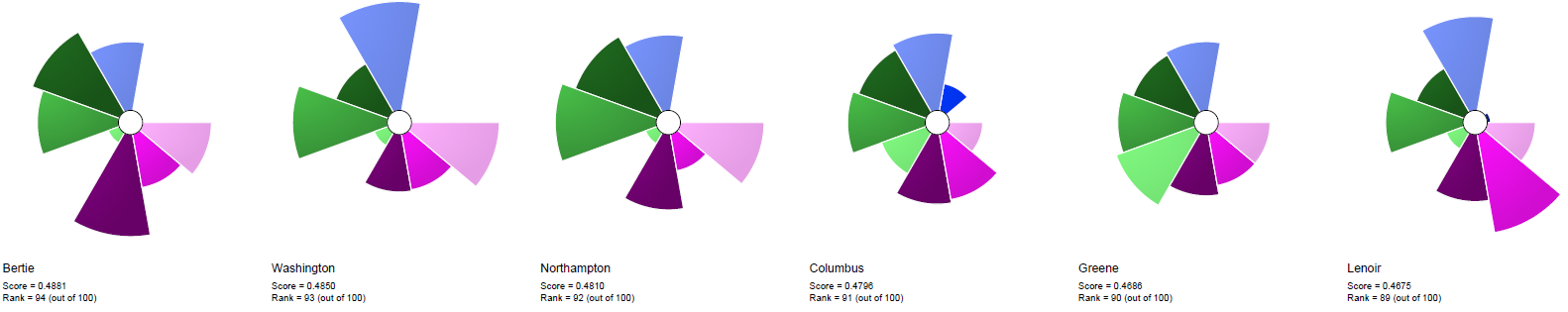  Columbus  Rank = 91 |
| 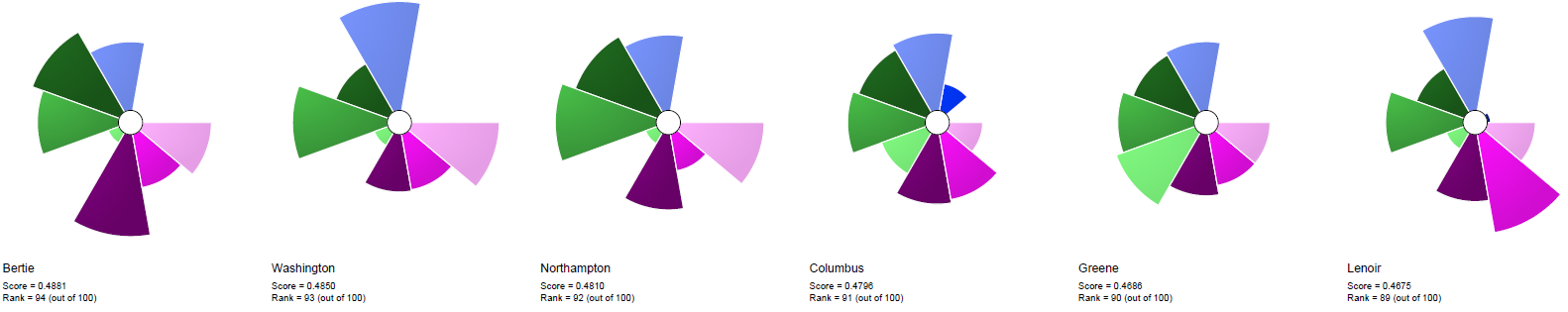  Greene  Rank = 90 | 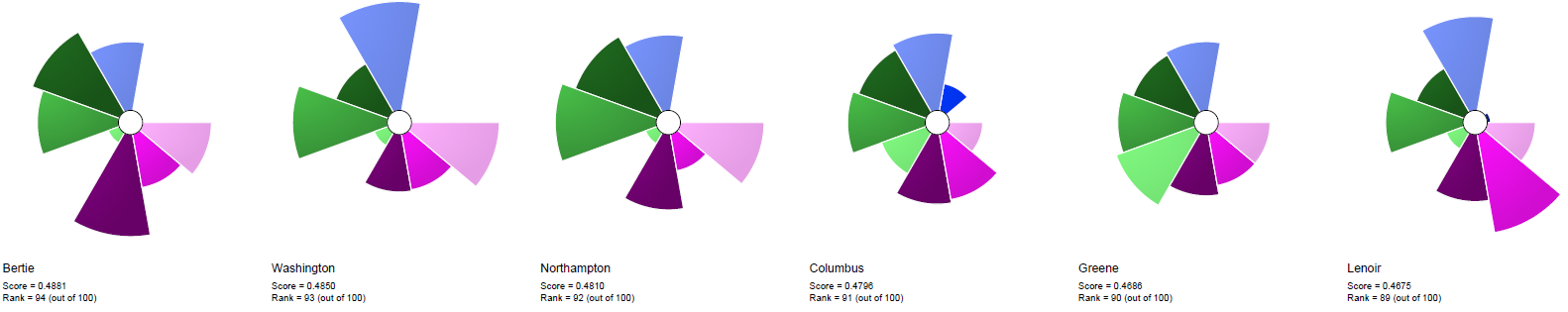  Lenoir  Rank = 89 | 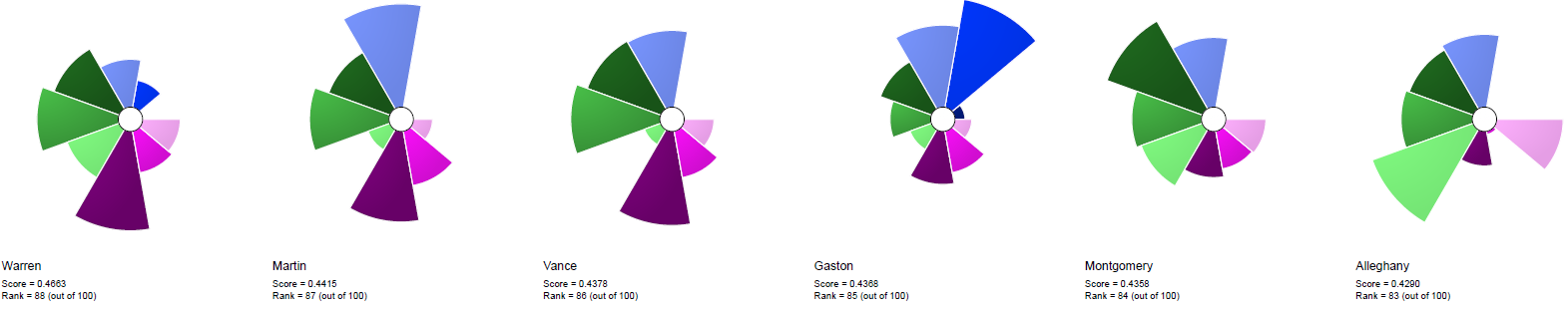  Warren  Rank = 88 | 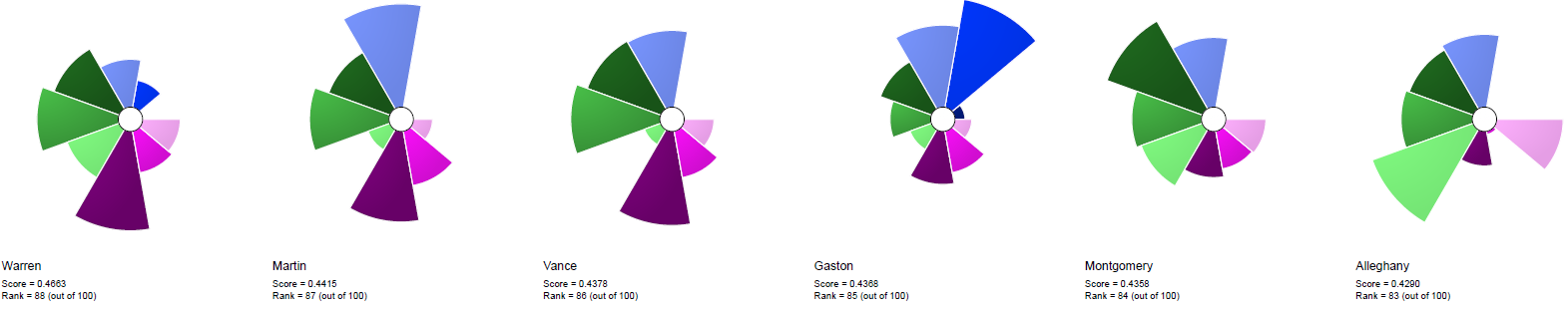  Martin  Rank = 87 | 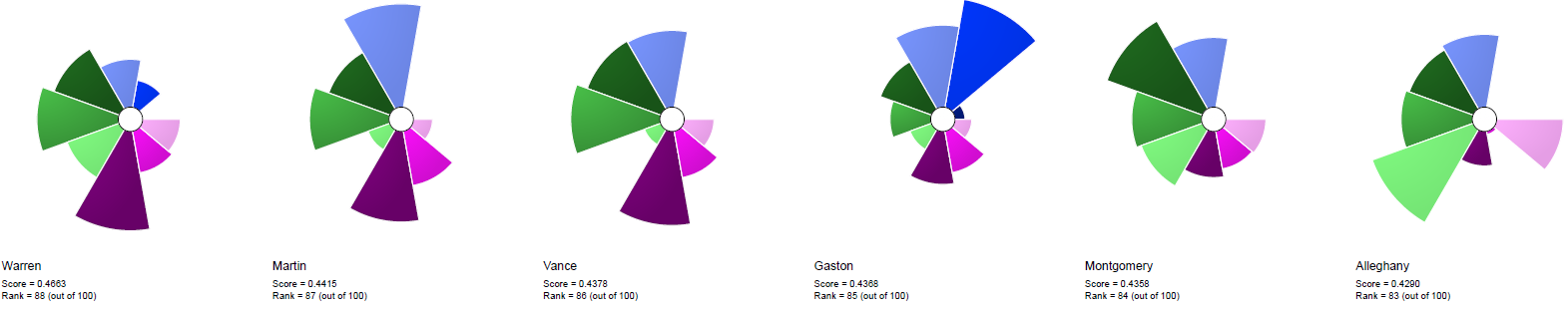  Vance  Rank = 86 |
| 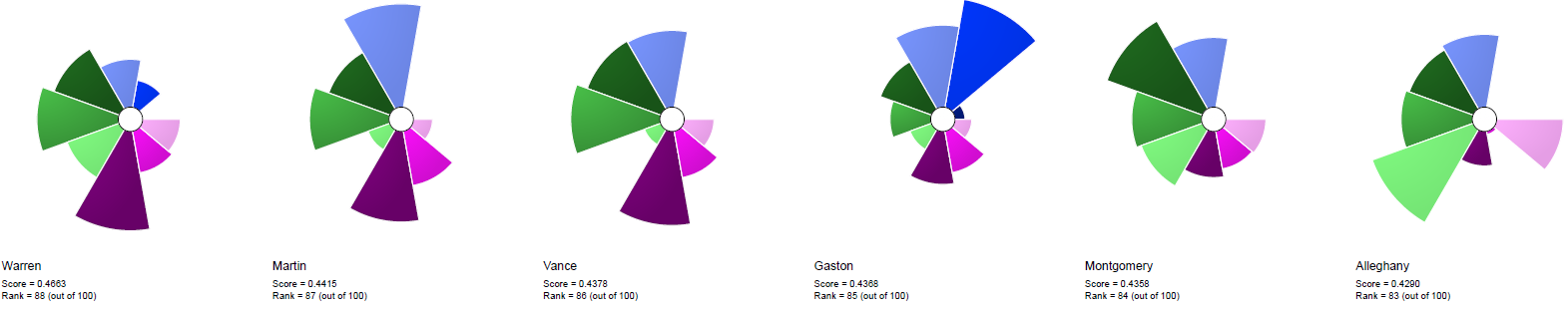  Gaston  Rank = 85 | 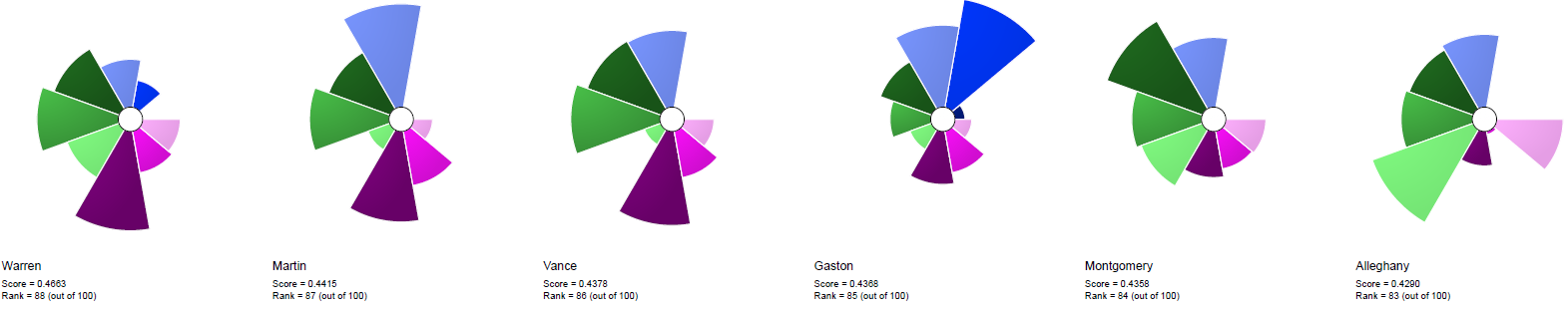  Montgomery  Rank = 84 | 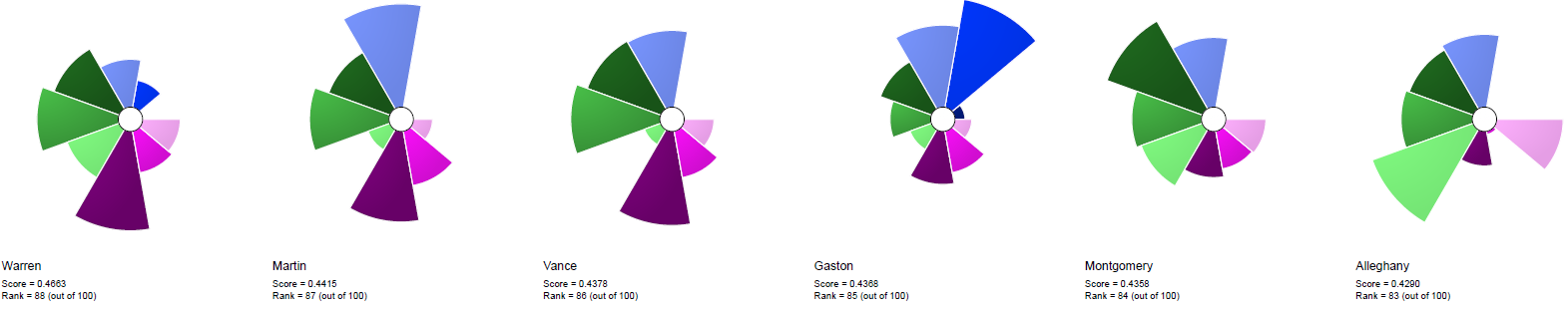  Alleghany  Rank = 83 | 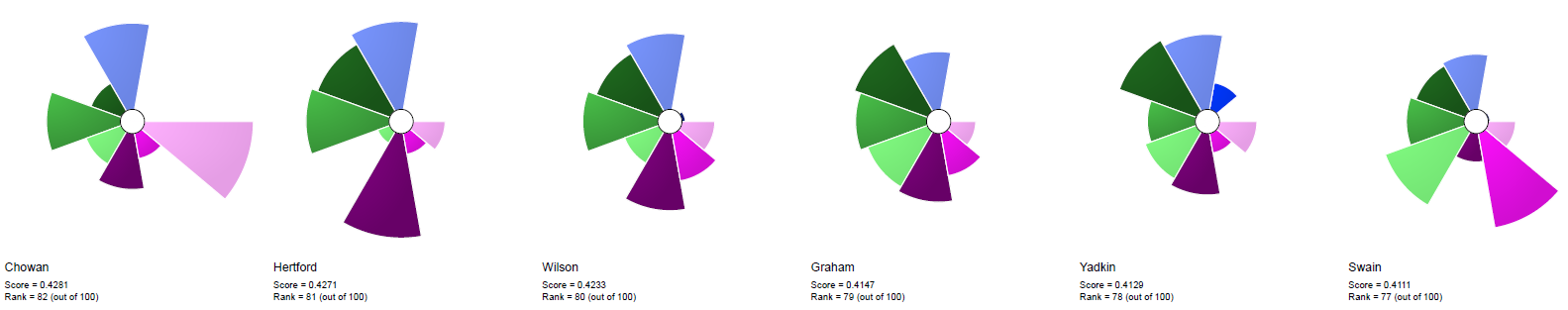  Chowan  Rank = 82 | 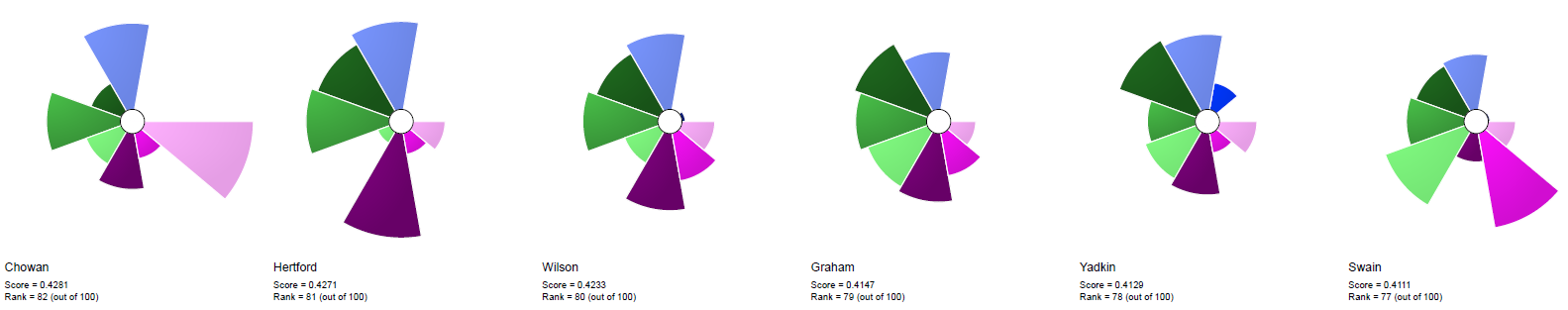  Hertford  Rank = 81 |
| 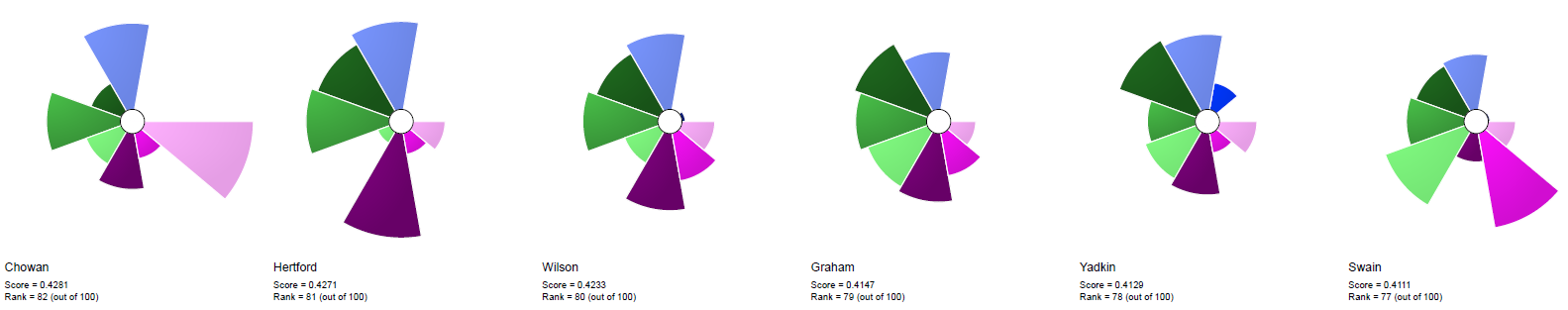  Wilson  Rank = 80 | 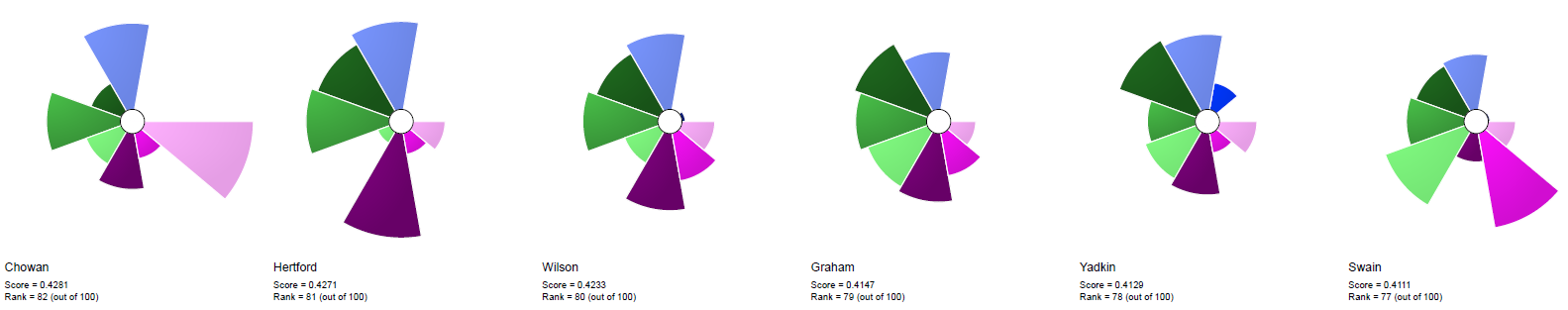  Graham  Rank = 79 | 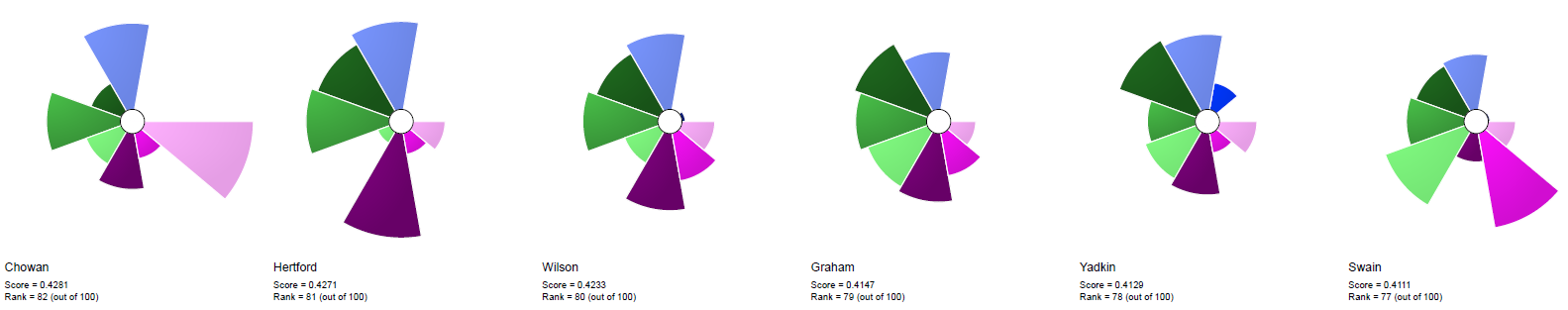  Yadkin  Rank = 78 | 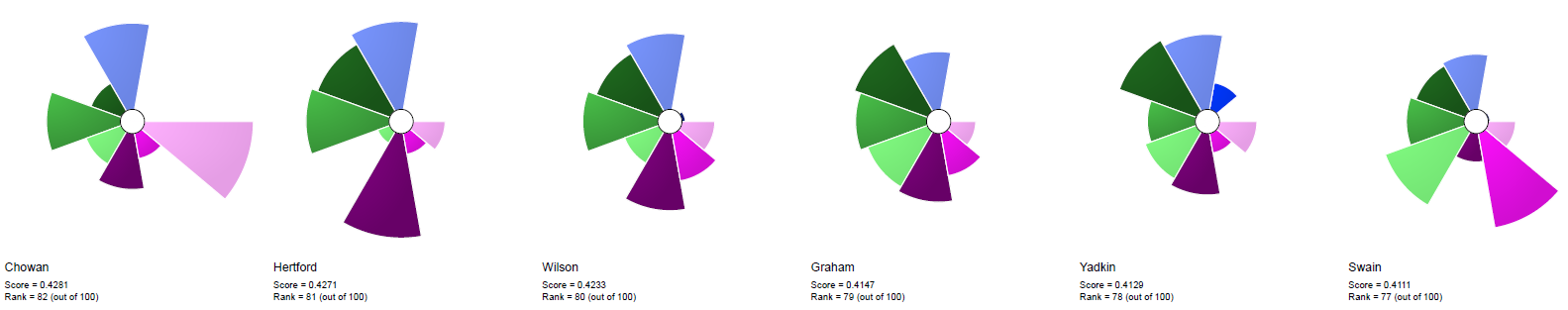  Swain  Rank = 77 | 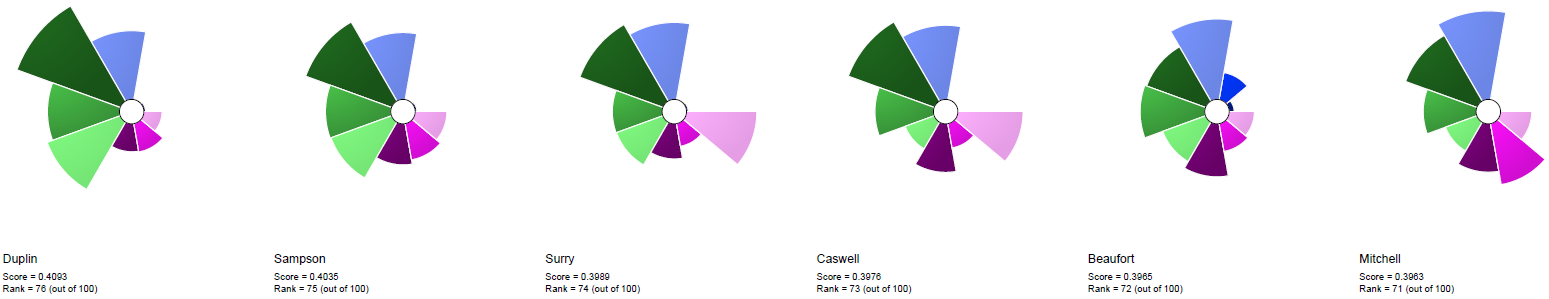  Duplin  Rank = 76 |
| 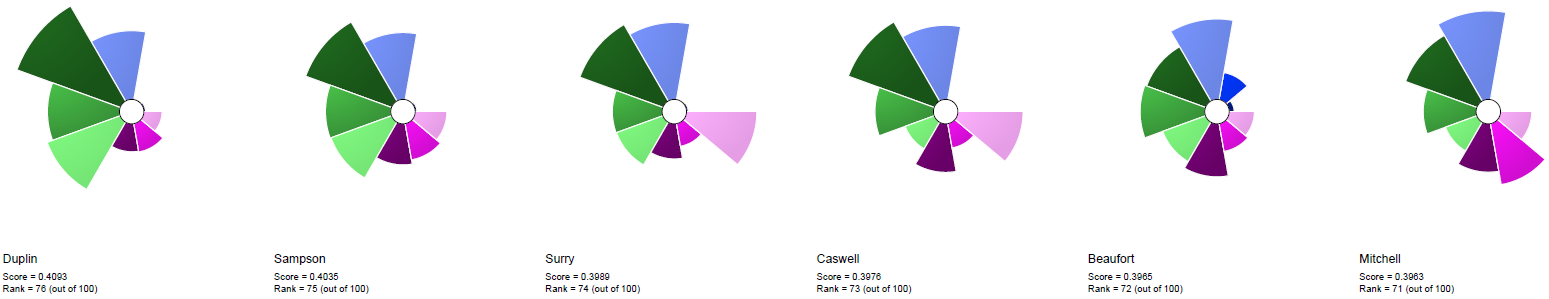  Sampson  Rank = 75 | 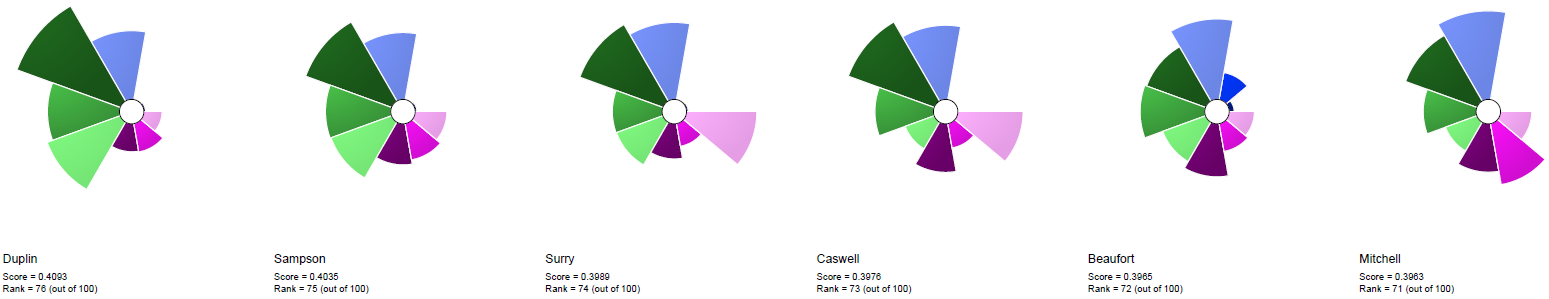  Surry  Rank = 74 | 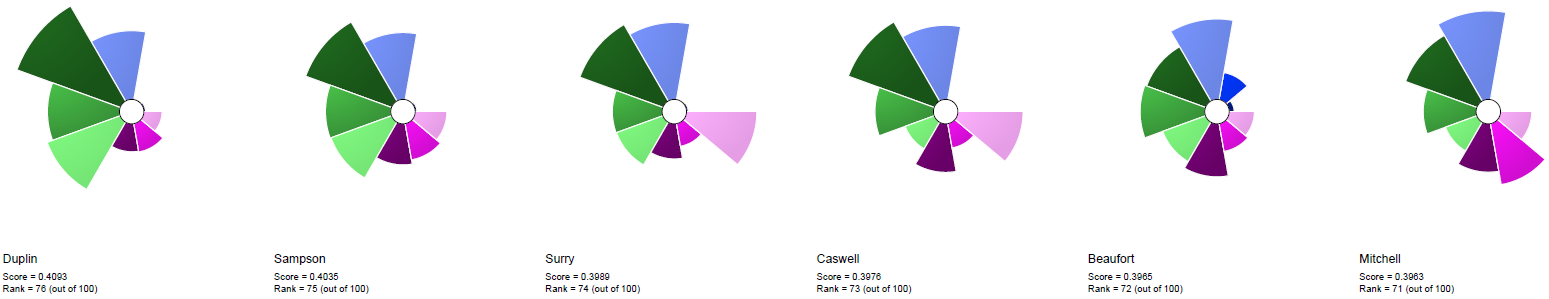  Caswell  Rank = 73 | 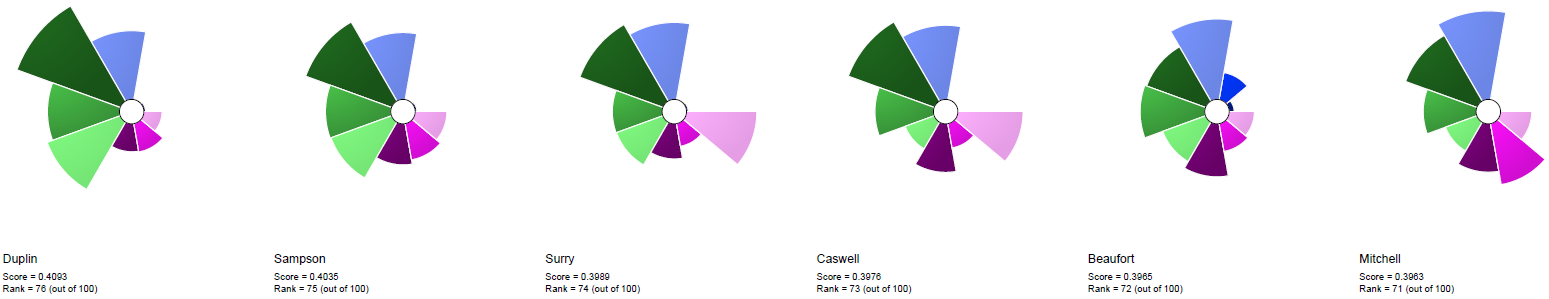  Beaufort  Rank = 72 | 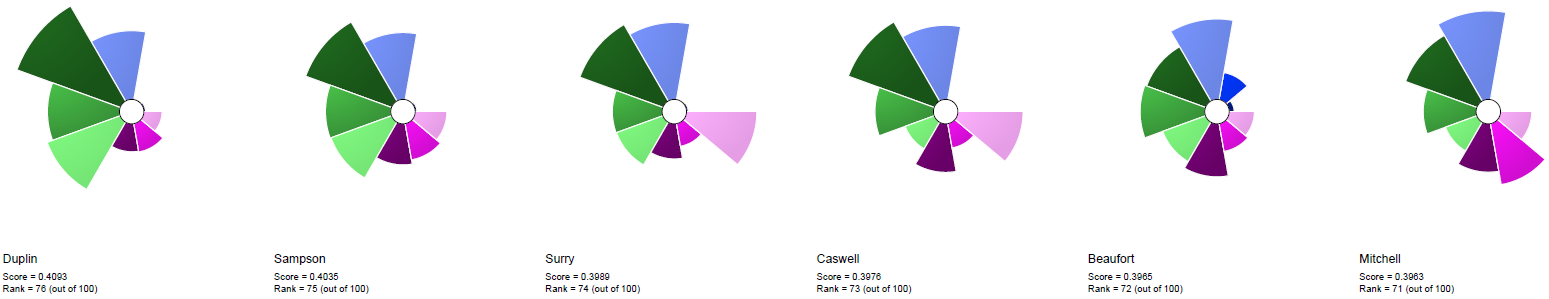  Mitchell  Rank = 71 |
| 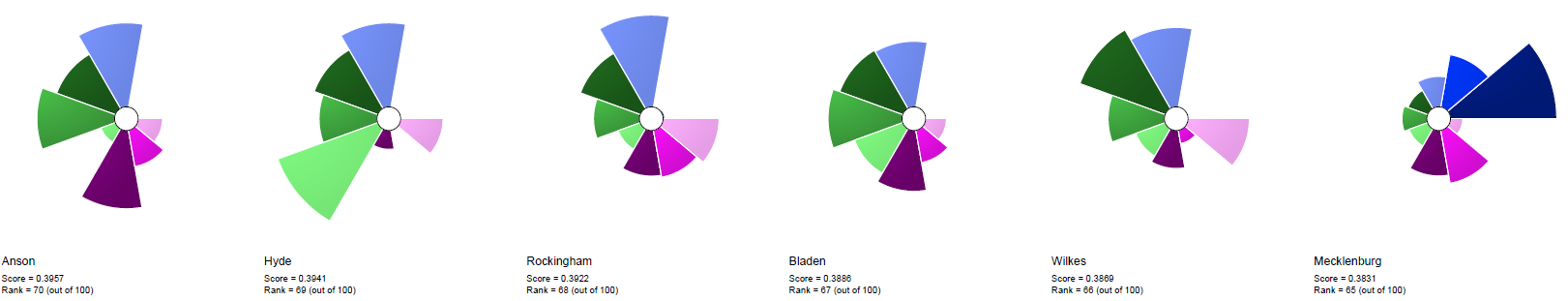  Anson  Rank = 70 | 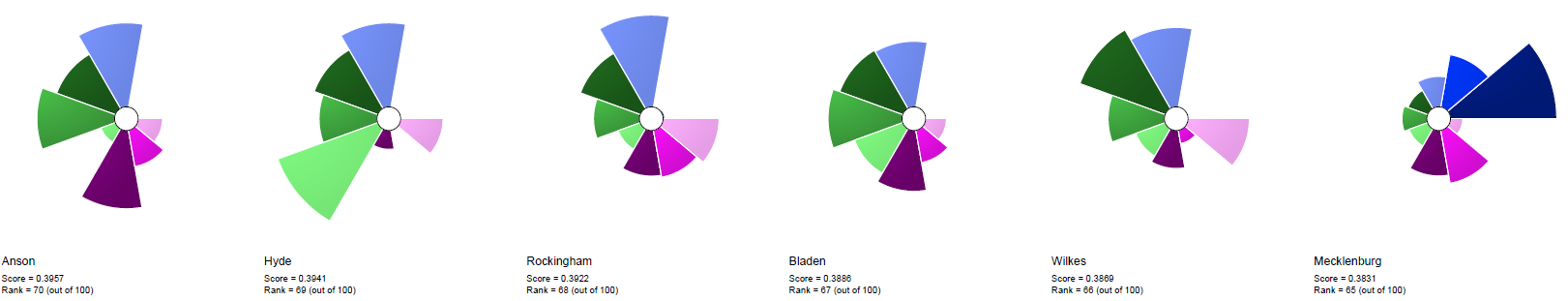  Hyde  Rank = 69 | 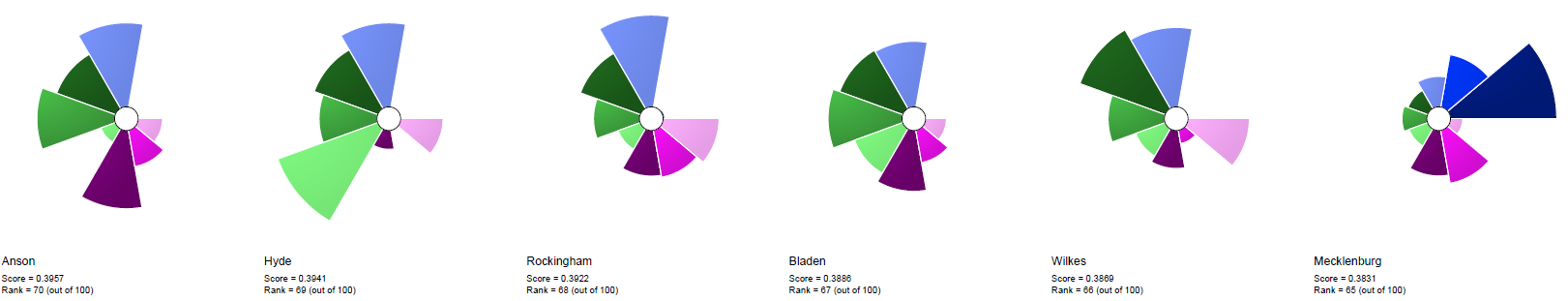  Rockingham  Rank = 68 | 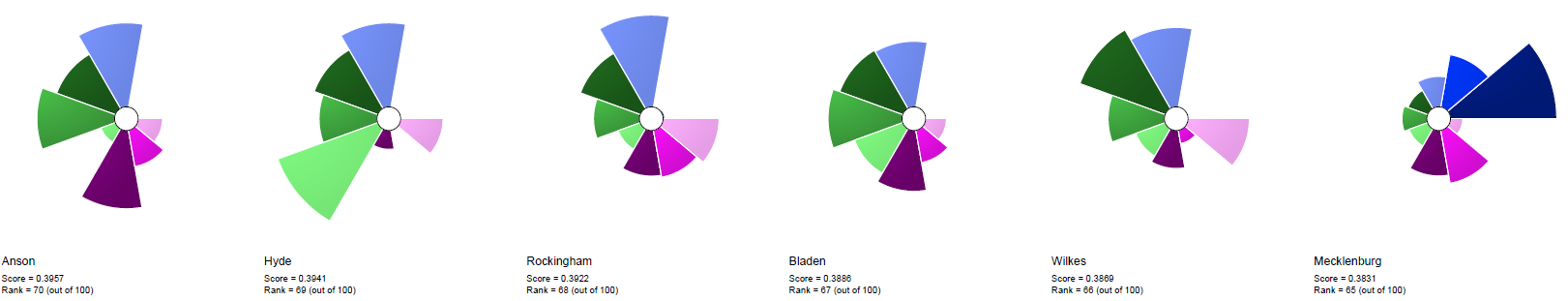  Bladen  Rank = 67 | 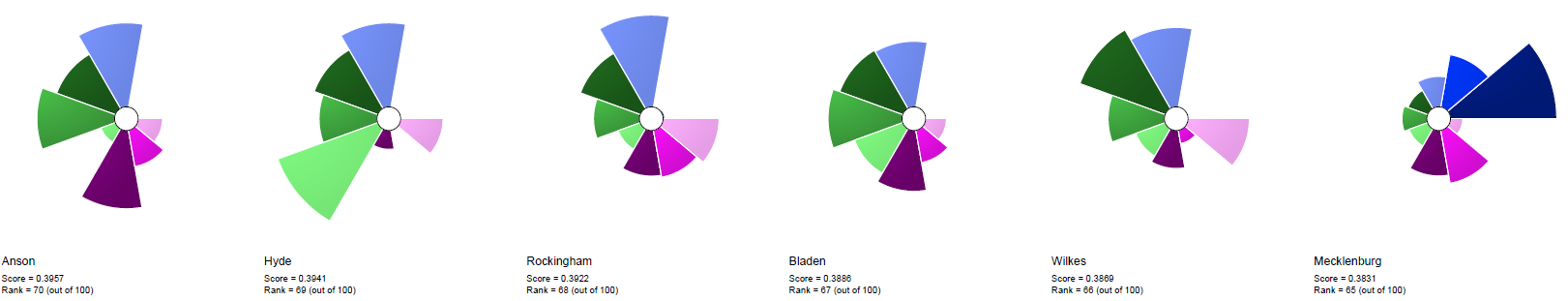  Wilkes  Rank = 66 |
| 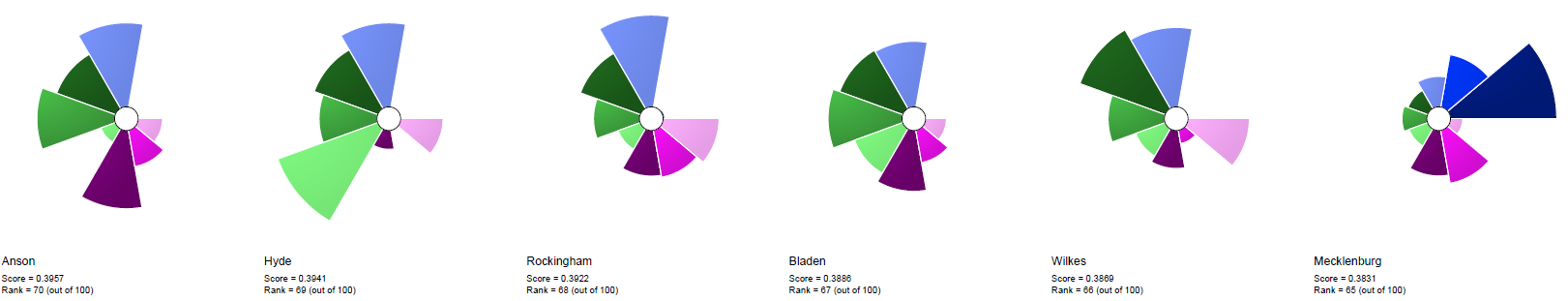  Mecklenburg  Rank = 65 | 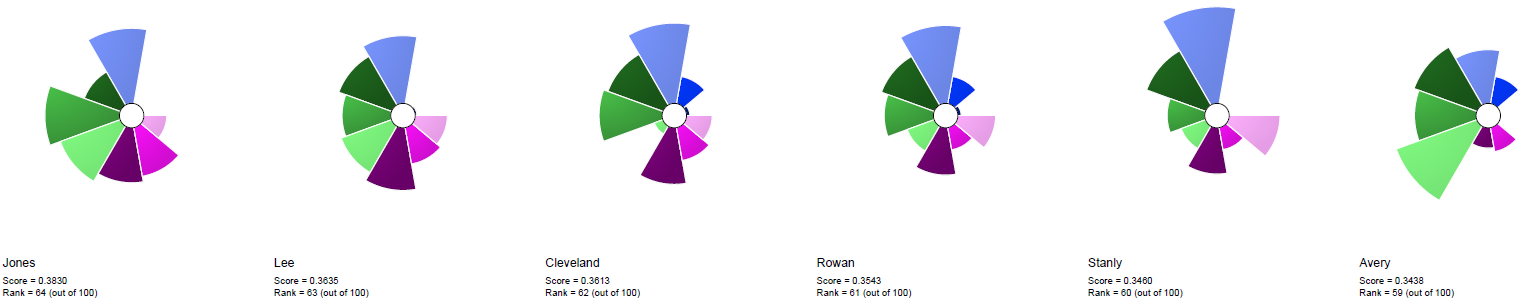  Jones  Rank = 64 | 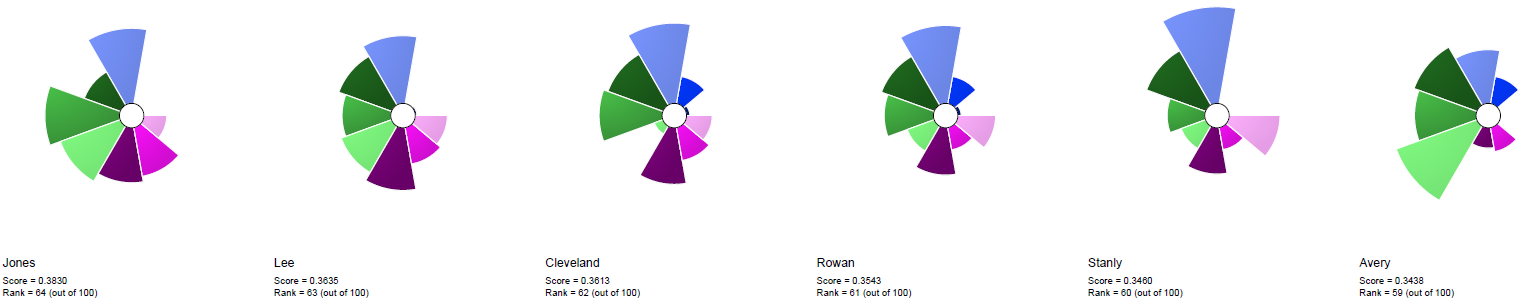  Lee  Rank = 63 | 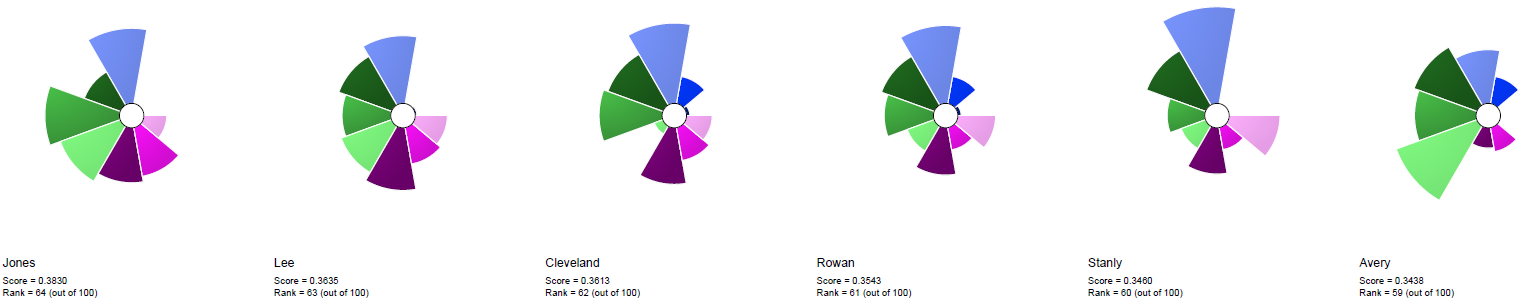  Cleveland  Rank = 62 | 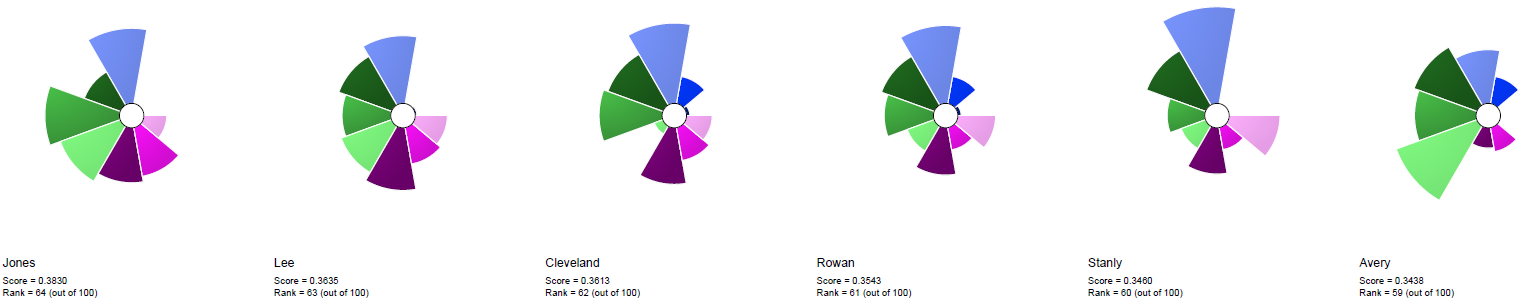  Rowan  Rank = 61 |
| 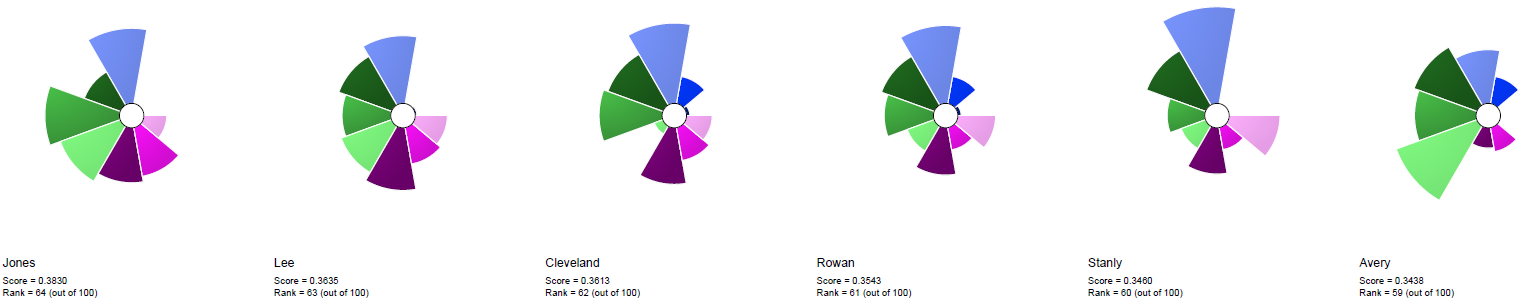  Stanly  Rank = 60 | 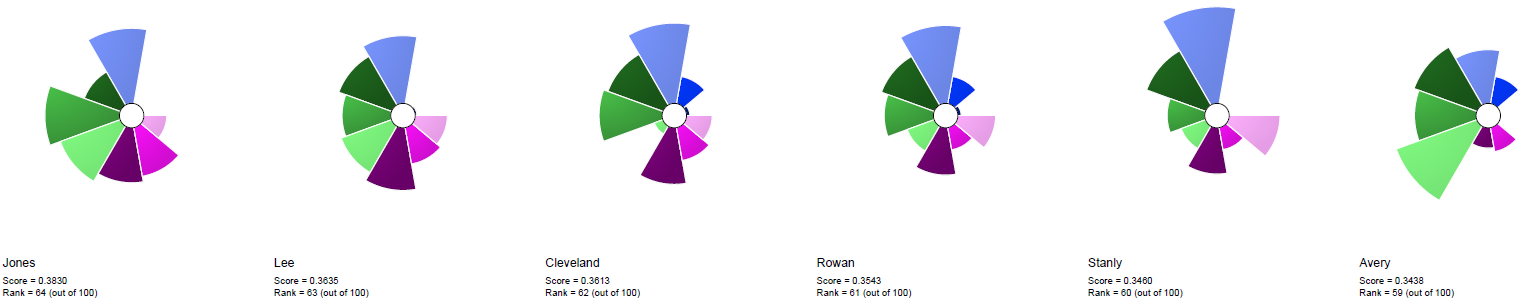  Avery  Rank = 59 | 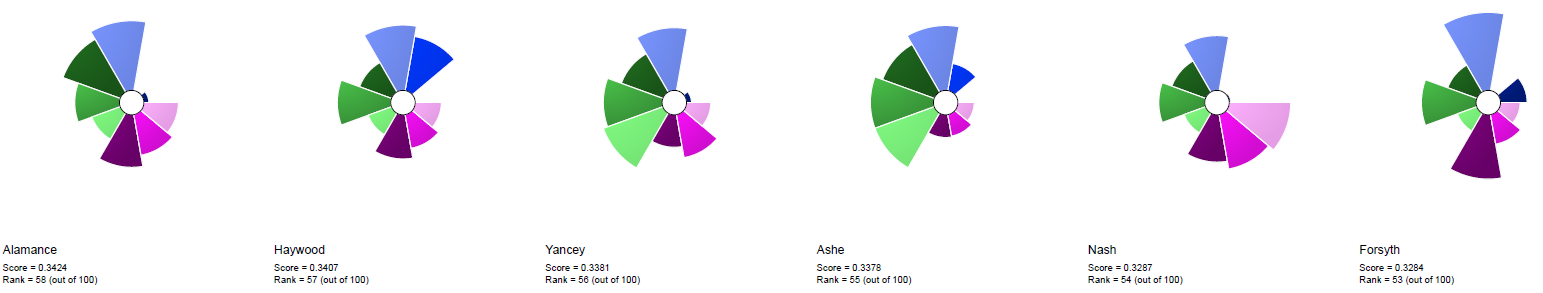  Alamance  Rank = 58 | 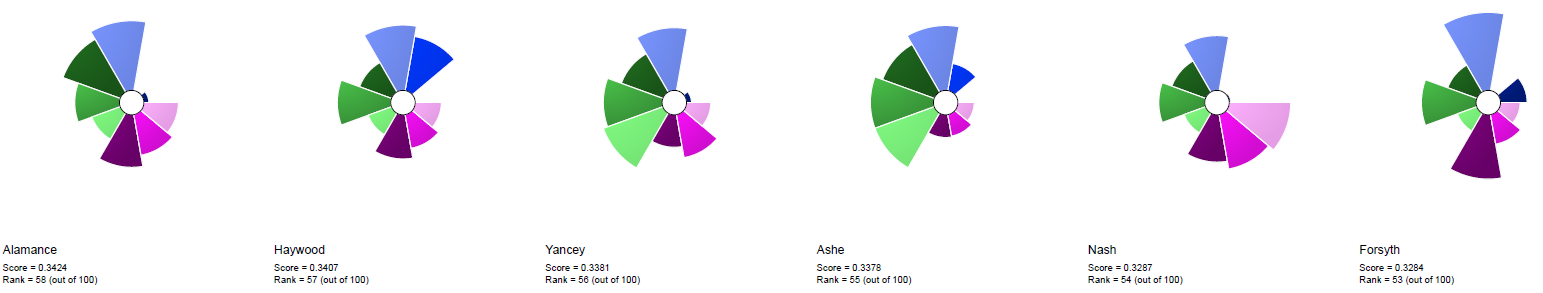  Haywood  Rank = 57 | 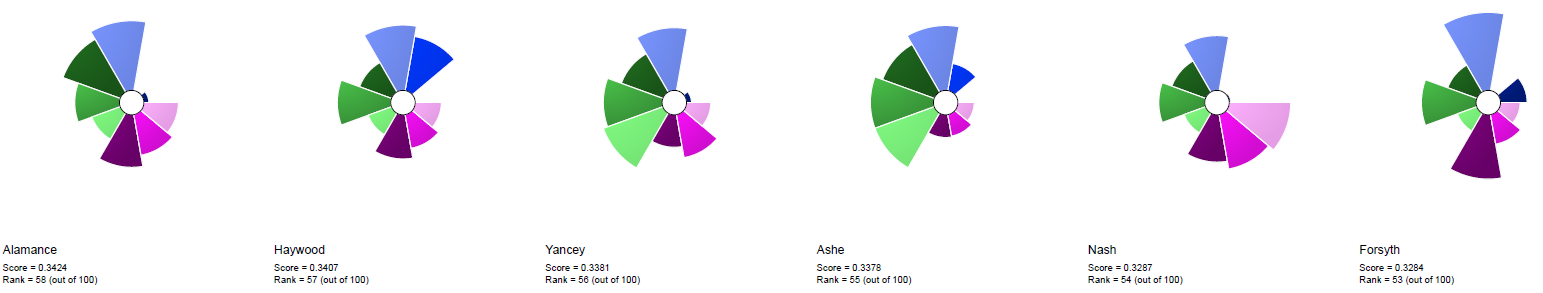  Yancey  Rank = 56 |
| 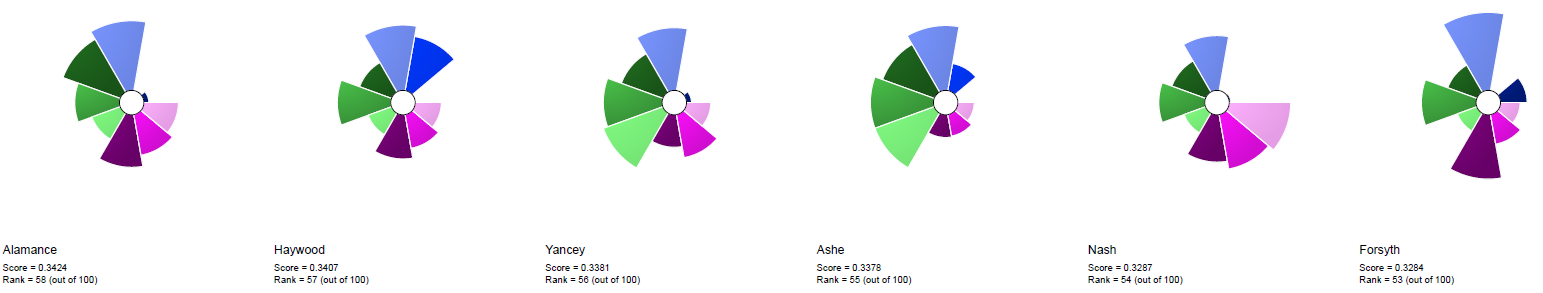  Ashe  Rank = 55 | 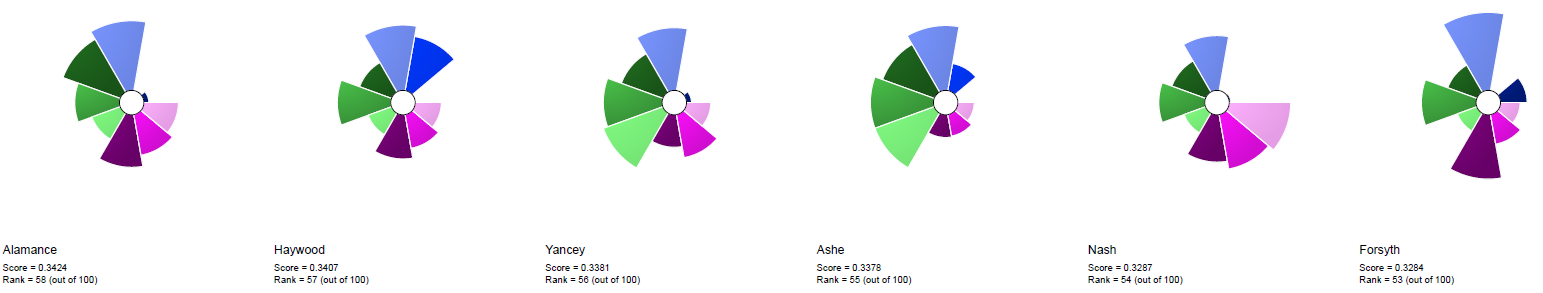  Nash  Rank = 54 | 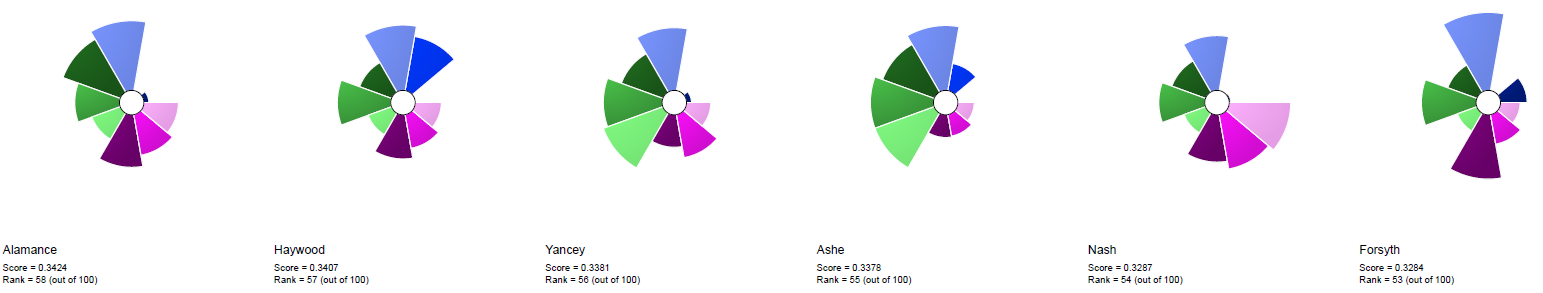  Forsyth  Rank = 53 | 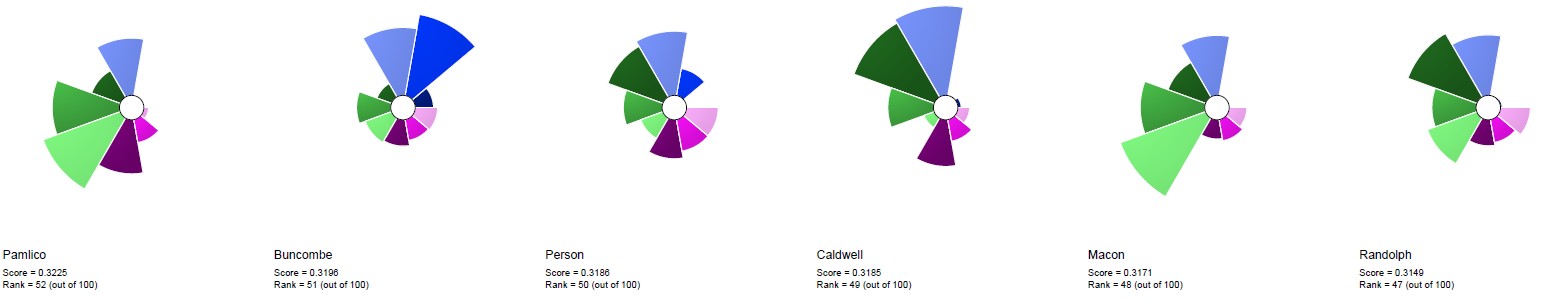  Pamlico  Rank = 52 | 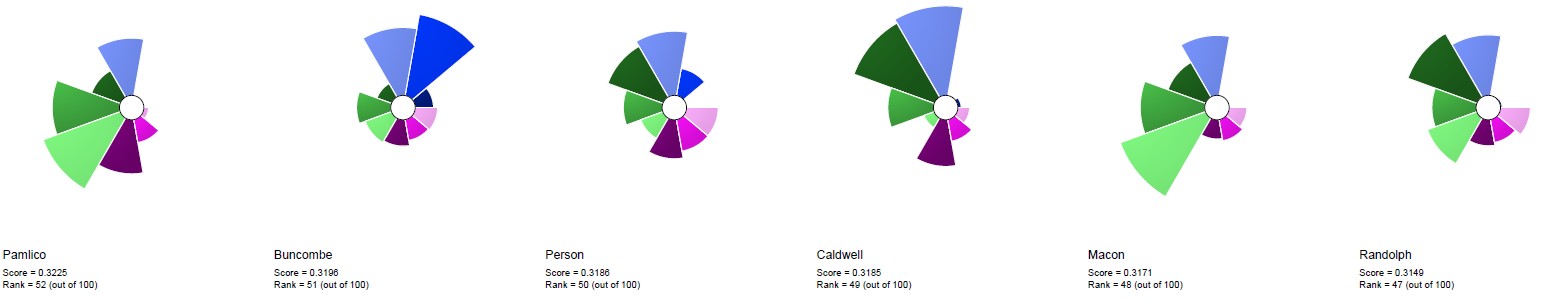  Buncombe  Rank = 51 |
| 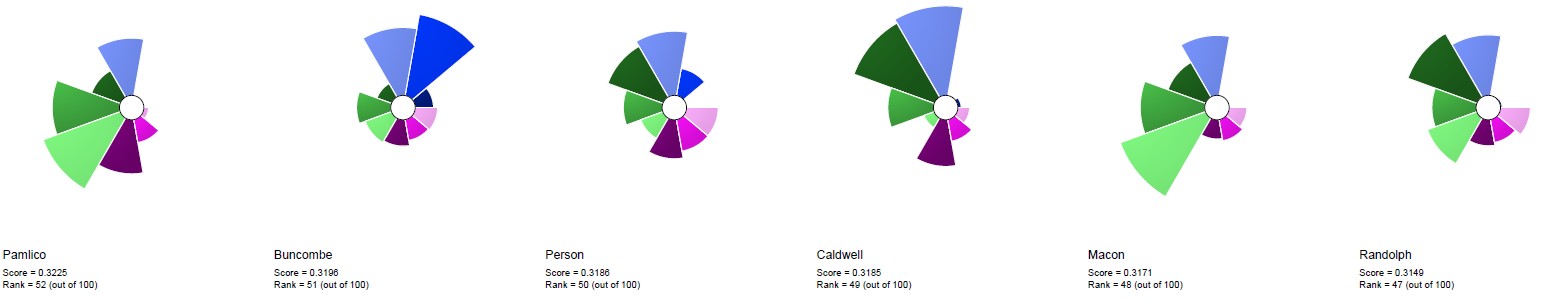  Person  Rank = 50 | 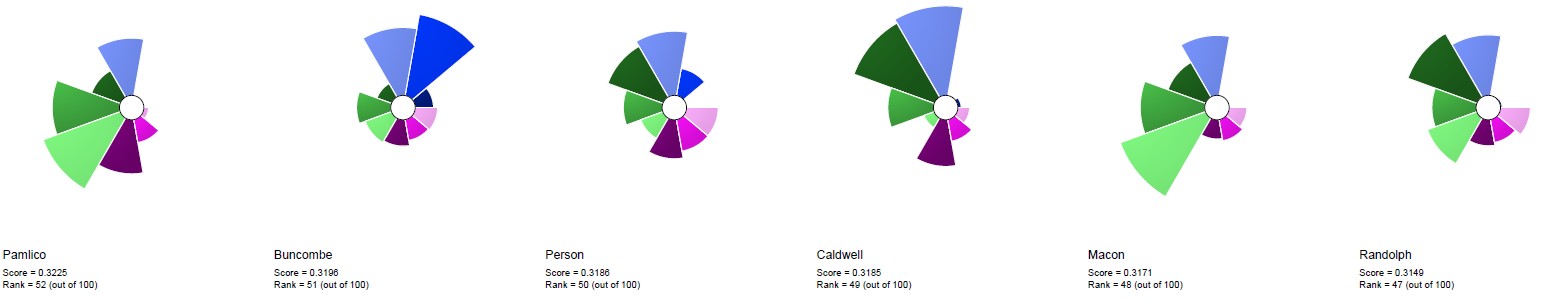  Caldwell  Rank = 49 | 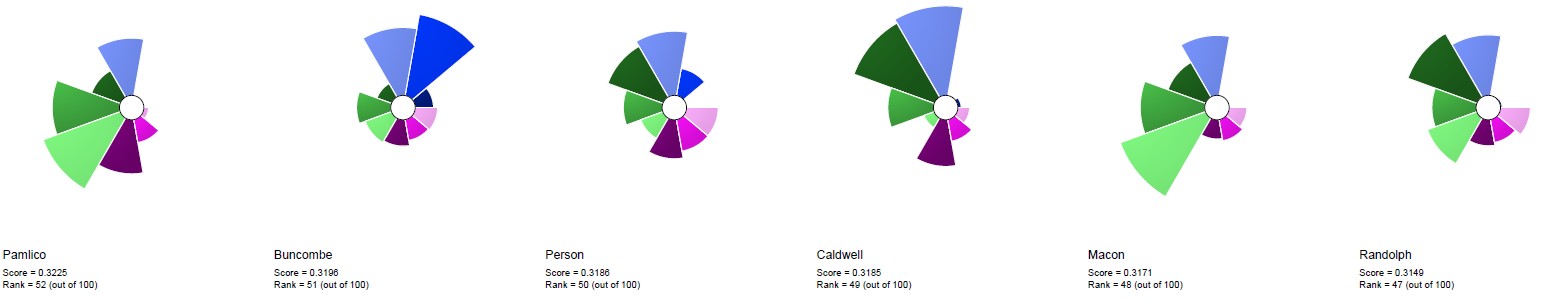  Macon  Rank = 48 | 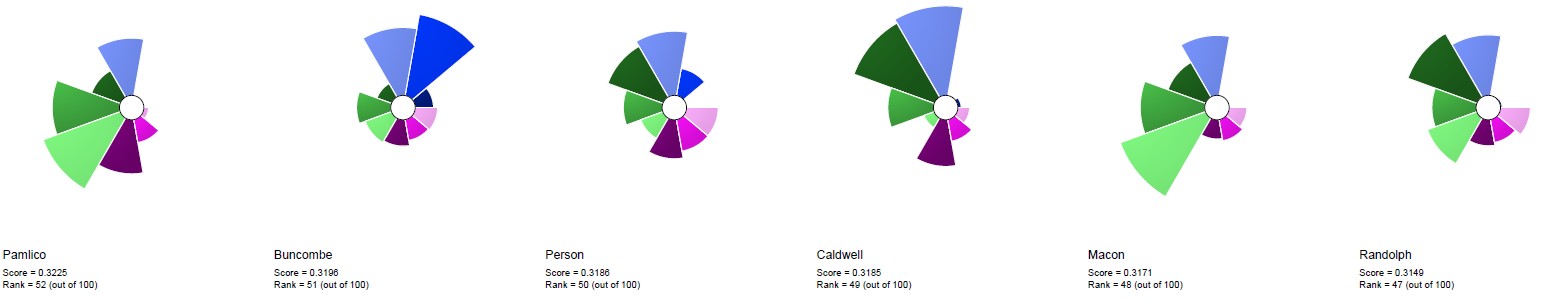  Randolph  Rank = 47 | 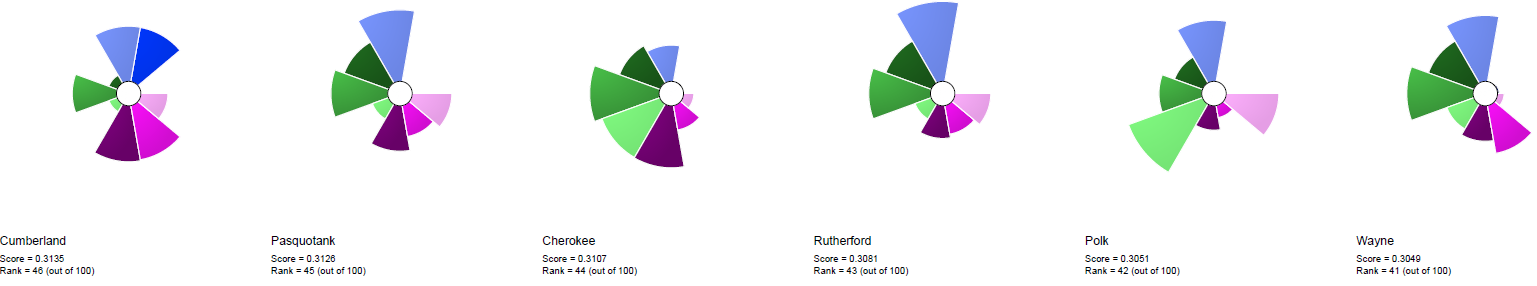  Cumberland  Rank = 46 |
| 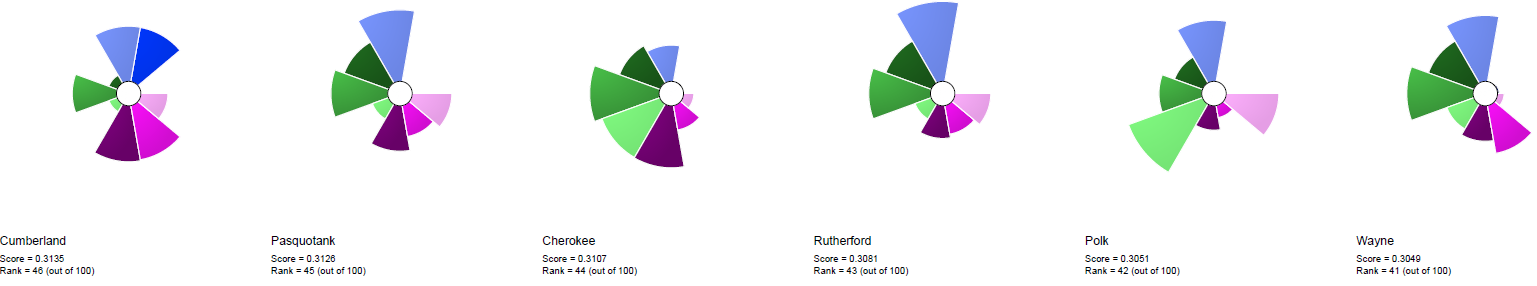  Pasquotank  Rank = 45 | 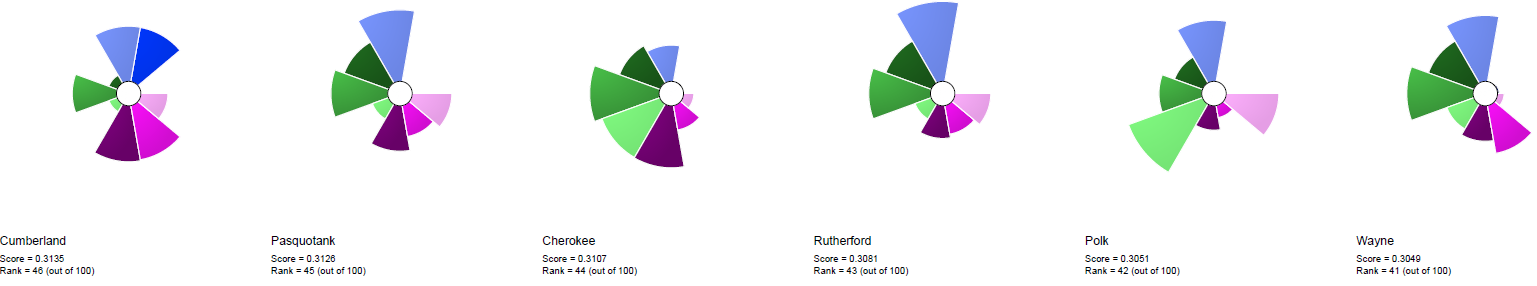  Cherokee  Rank = 44 | 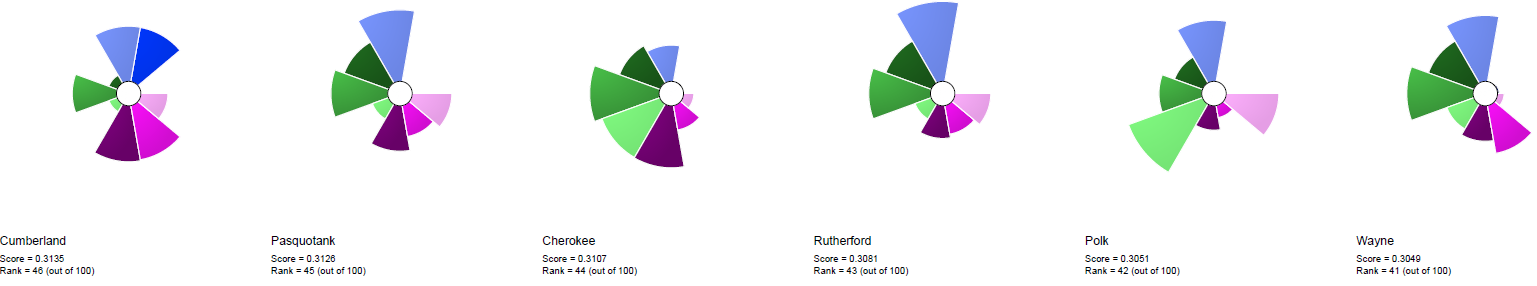  Rutherford  Rank = 43 | 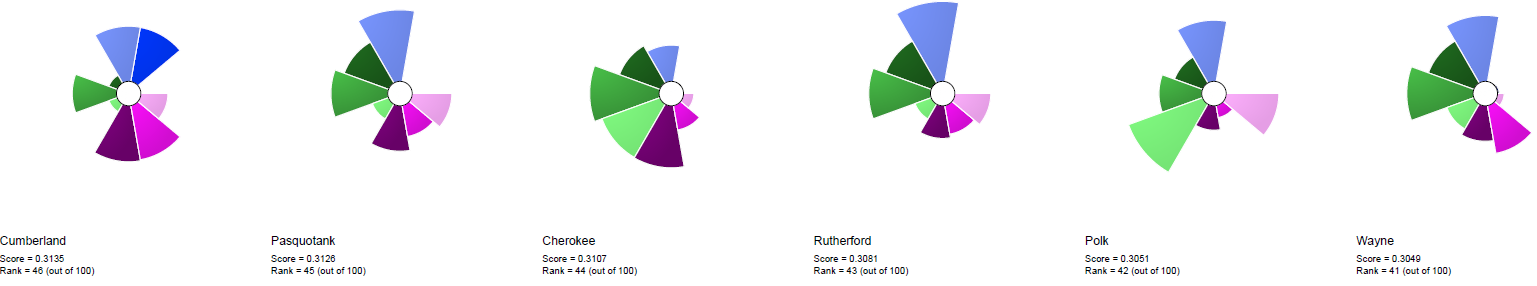  Polk  Rank = 42 | 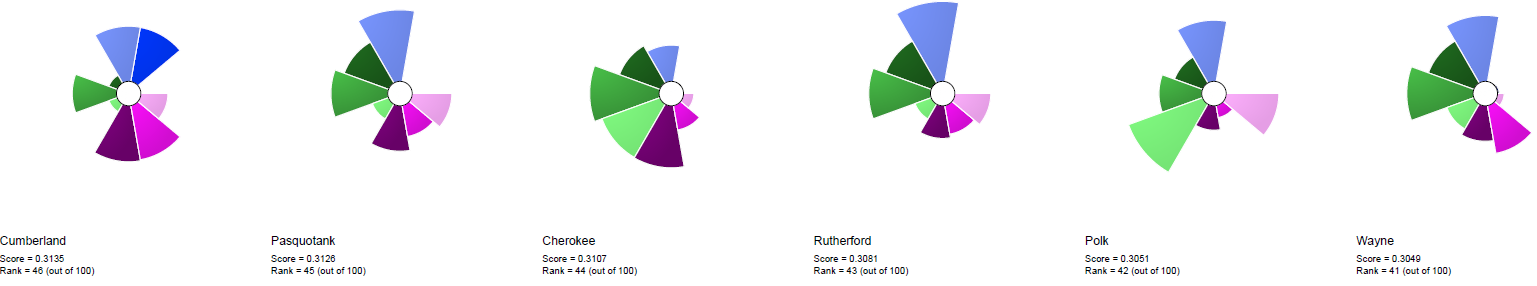  Wayne  Rank = 41 |
| 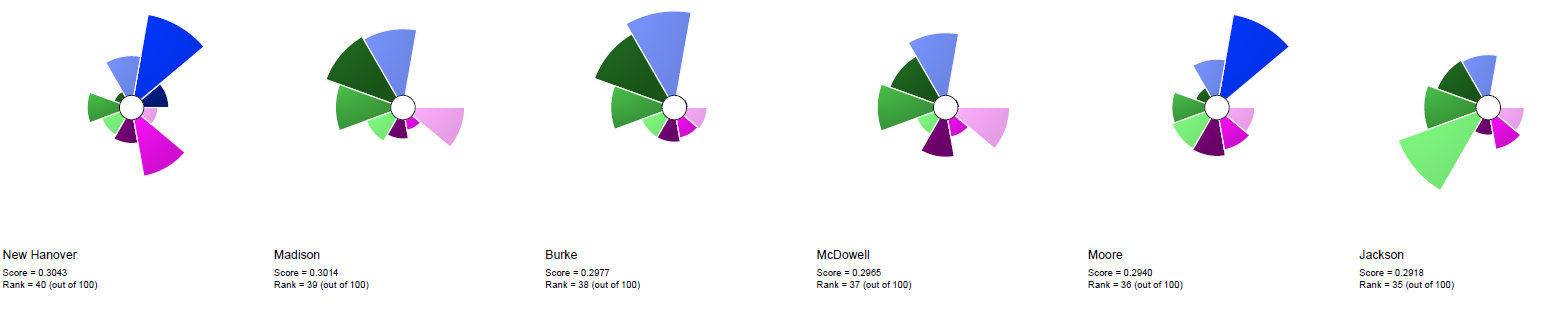  New Hanover  Rank = 40 | 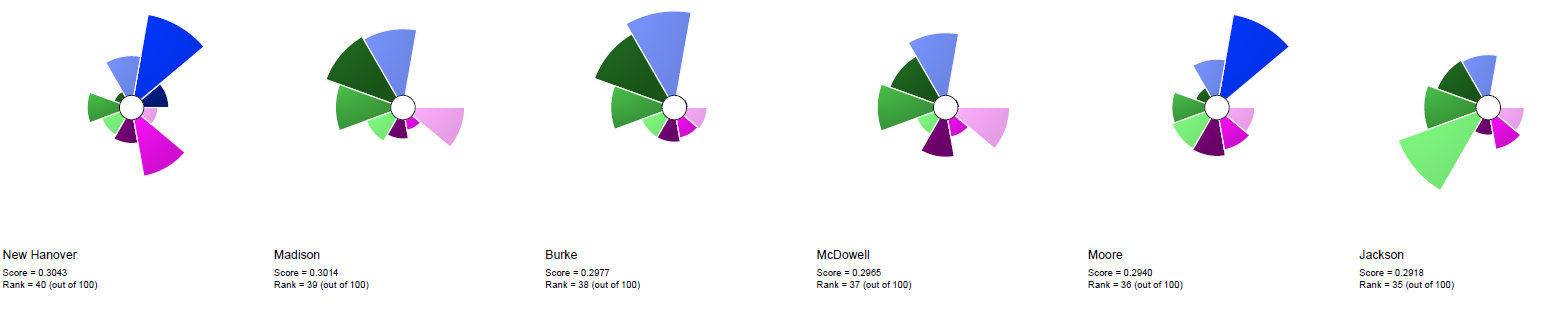  Madison  Rank = 39 | 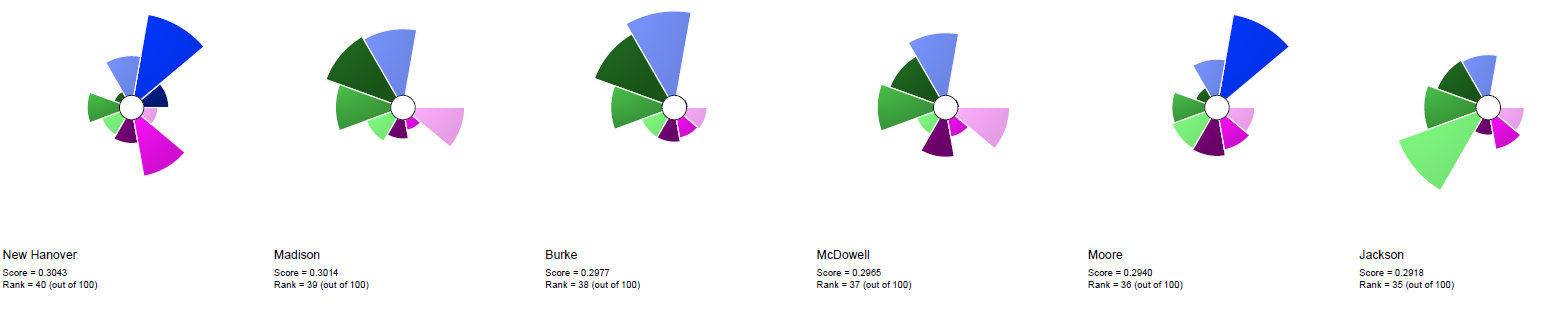  Burke  Rank = 38 | 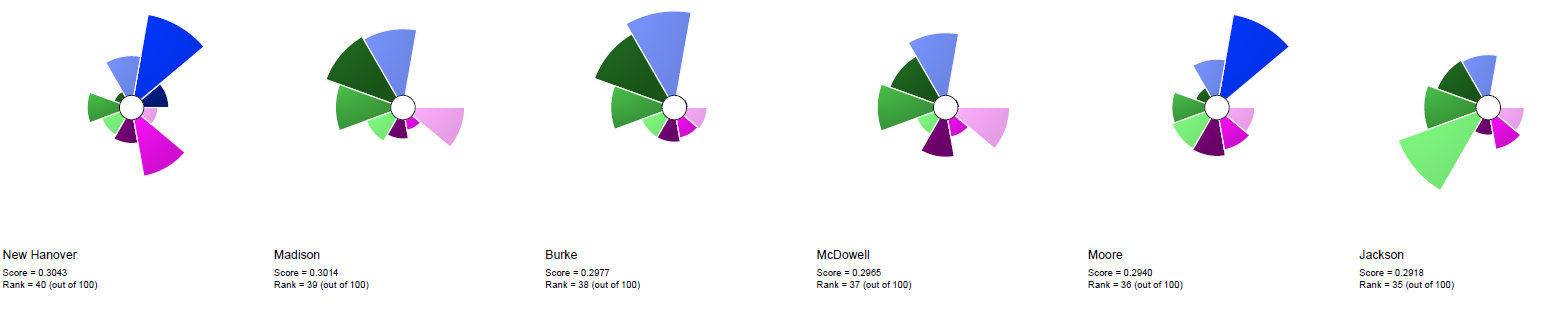  McDowell  Rank = 37 | 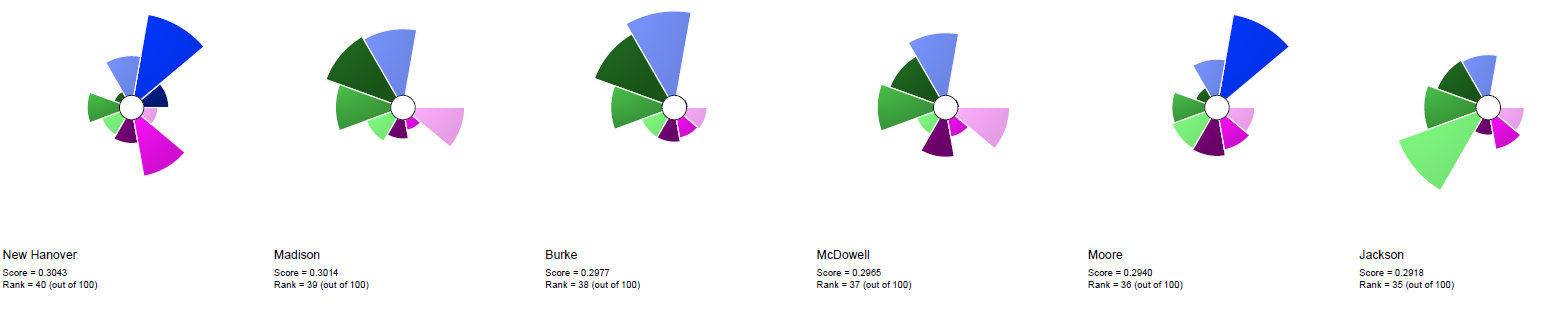  Moore  Rank = 36 |
| 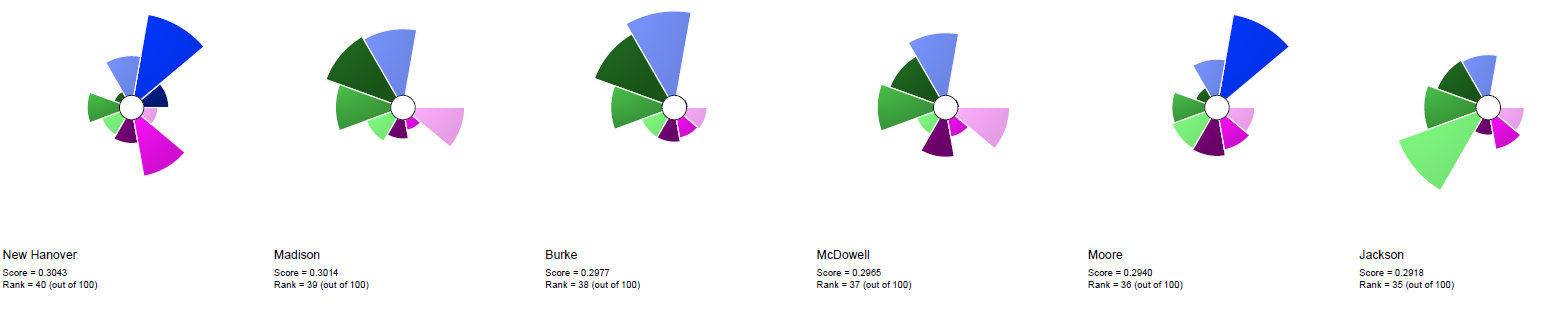  Jackson  Rank = 35 | 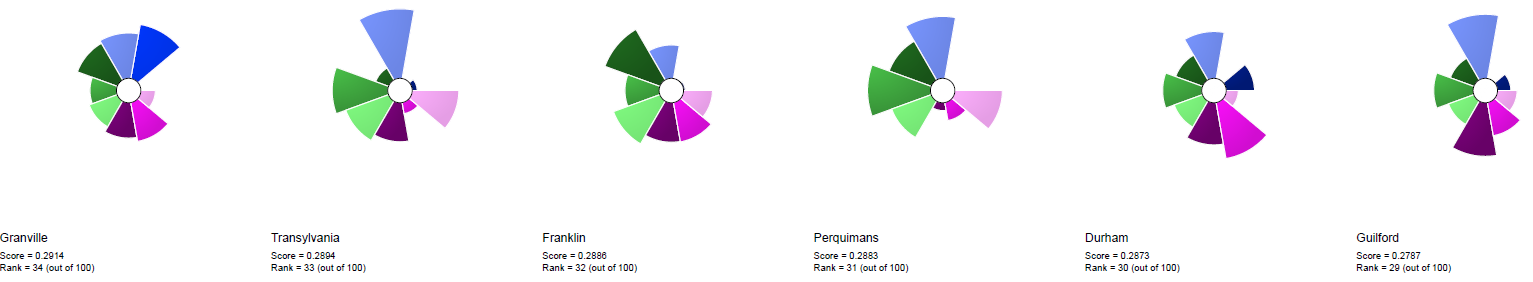  Granville  Rank = 34 | 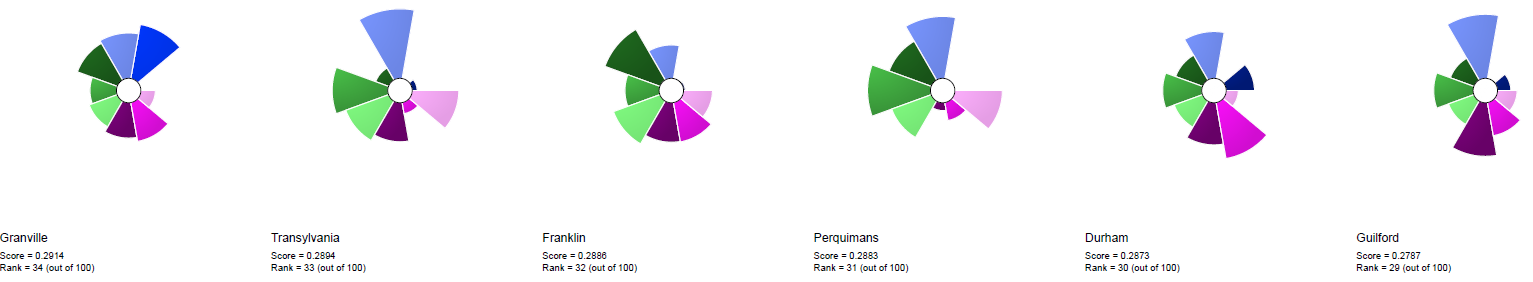  Transylvania  Rank = 33 | 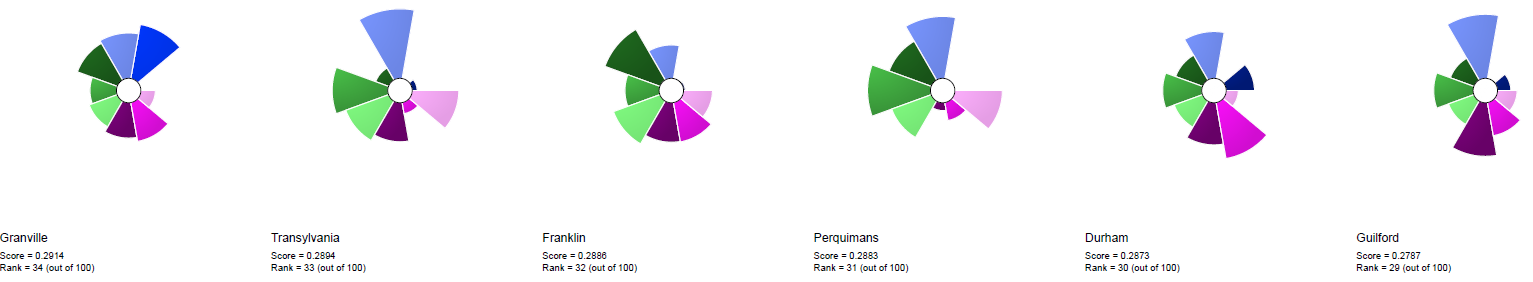  Franklin  Rank = 32 | 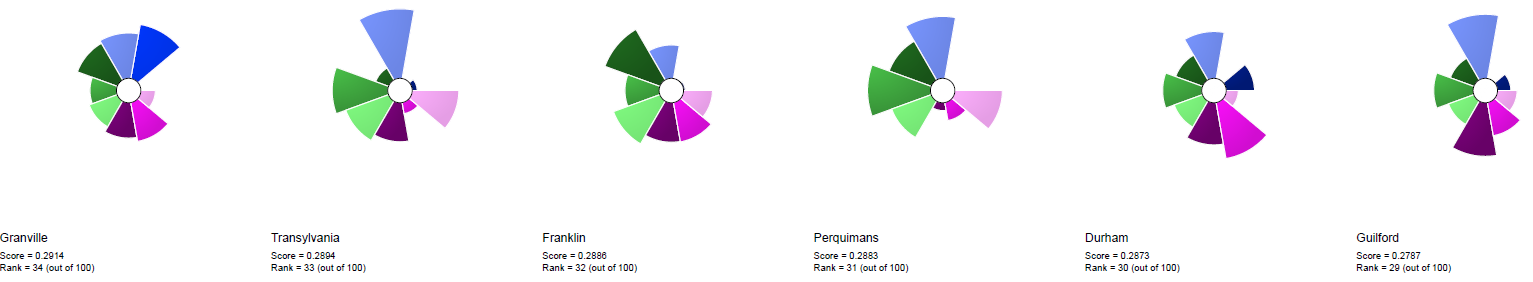  Perquimans  Rank = 31 |
| 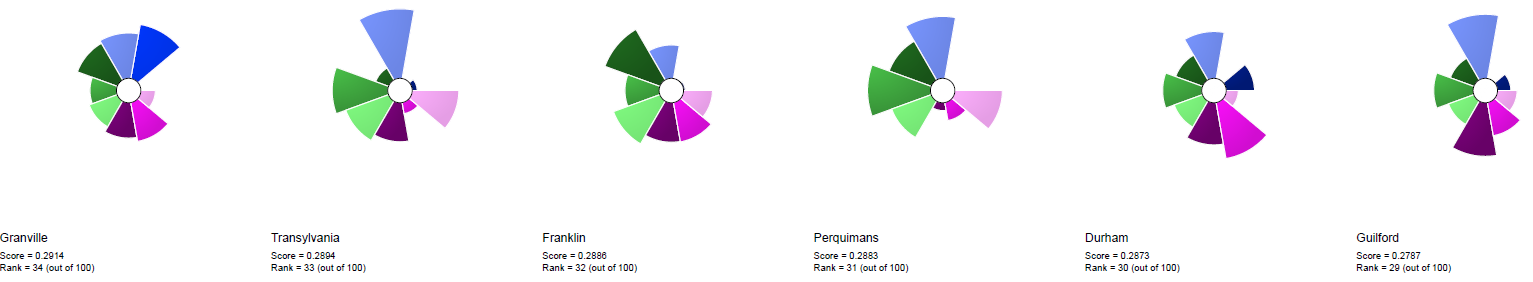  Durham  Rank = 30 | 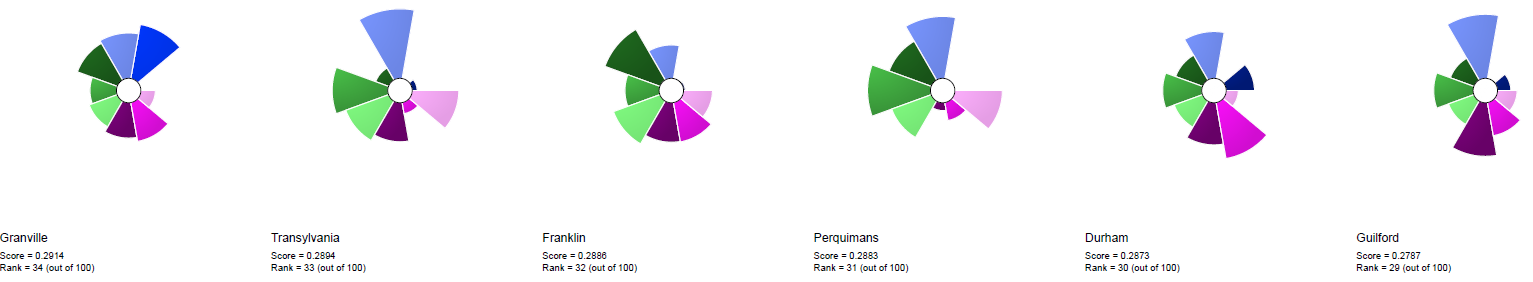  Guilford  Rank = 29 | 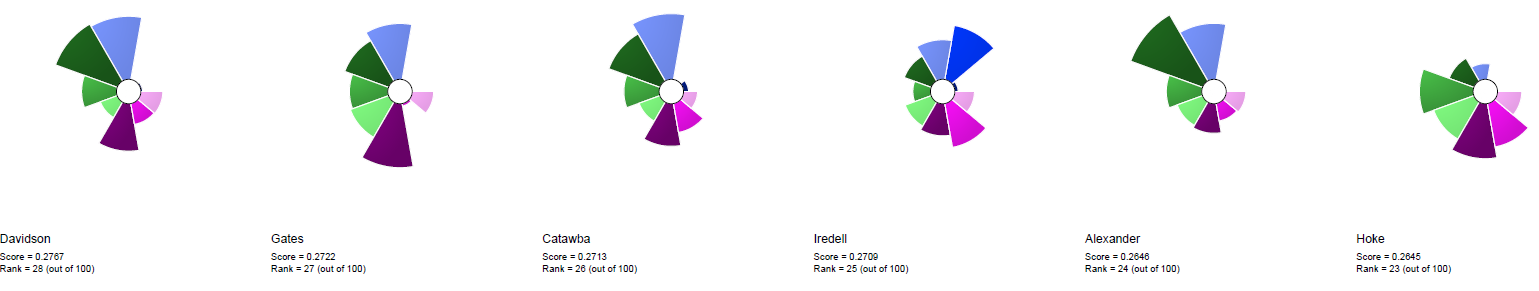  Davidson  Rank = 28 | 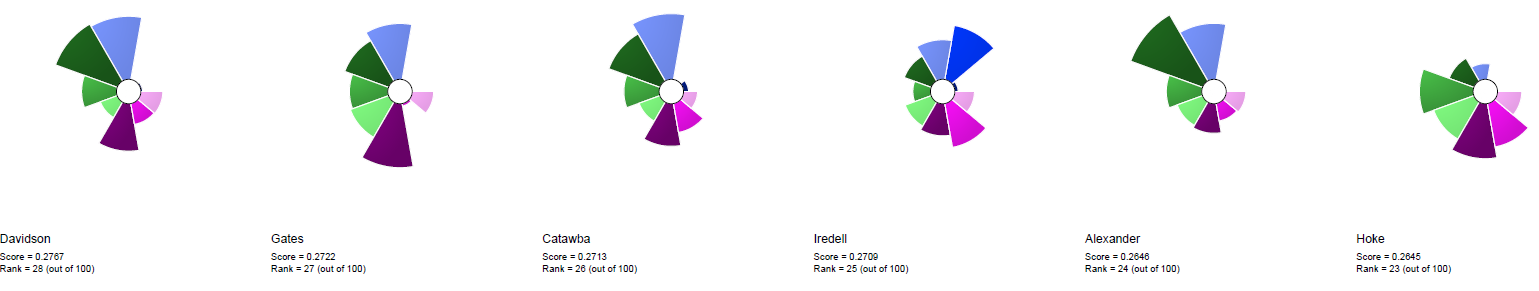  Gates  Rank = 27 | 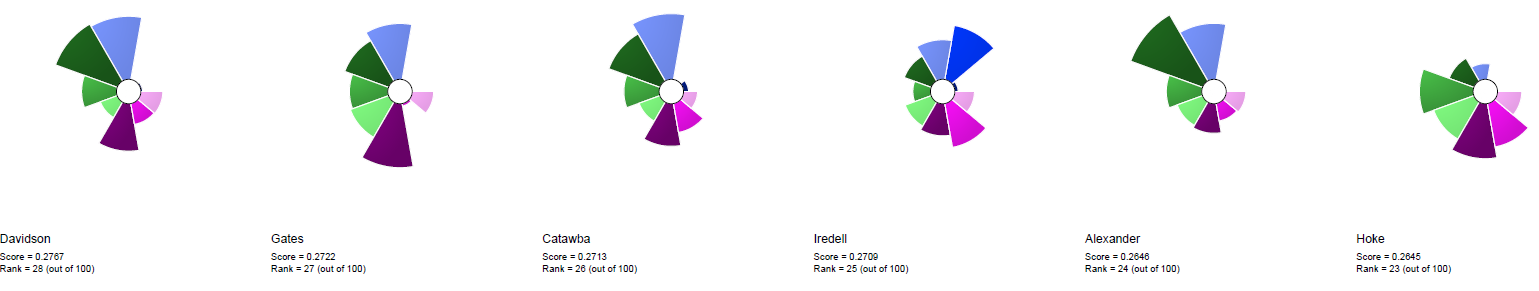  Catawba  Rank = 26 |
| 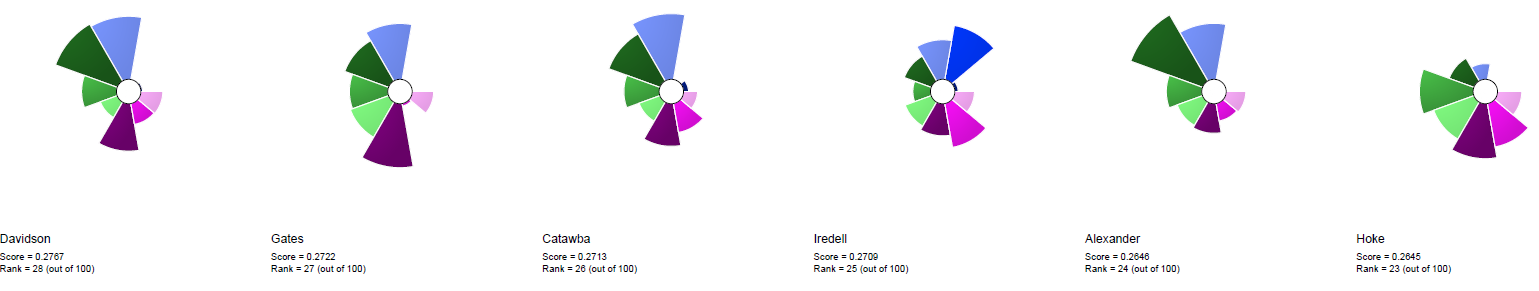  Iredell  Rank = 25 | 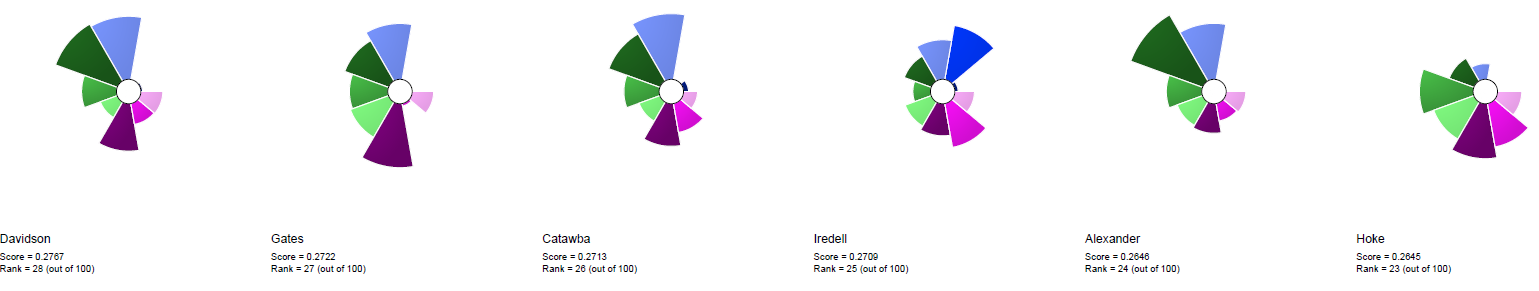  Alexander  Rank = 24 | 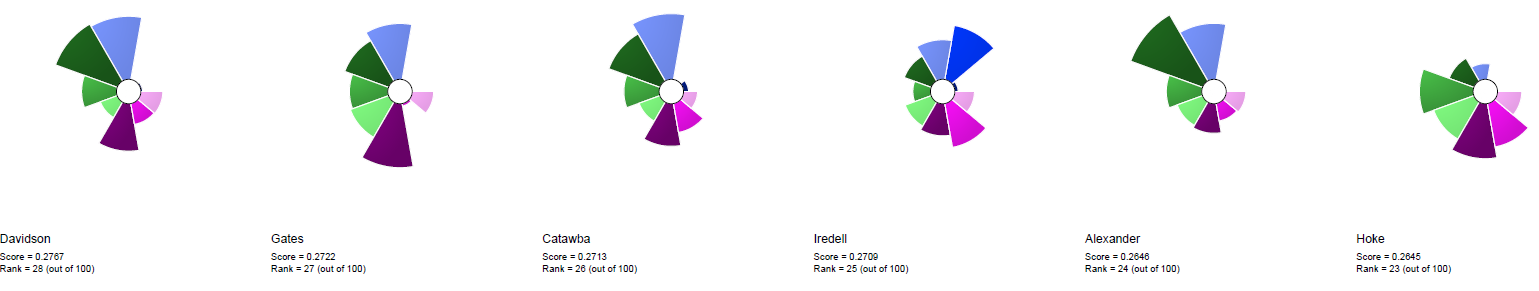  Hoke  Rank = 23 | 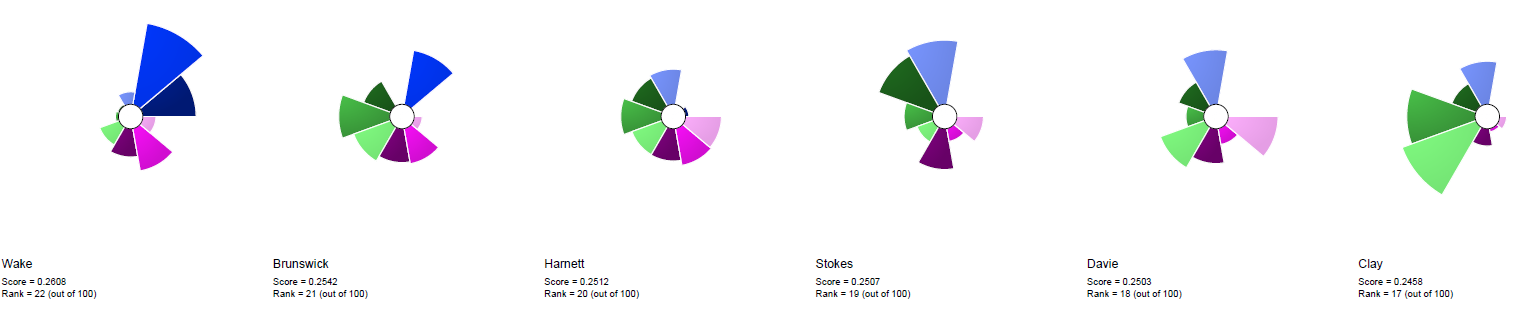  Wake  Rank = 22 | 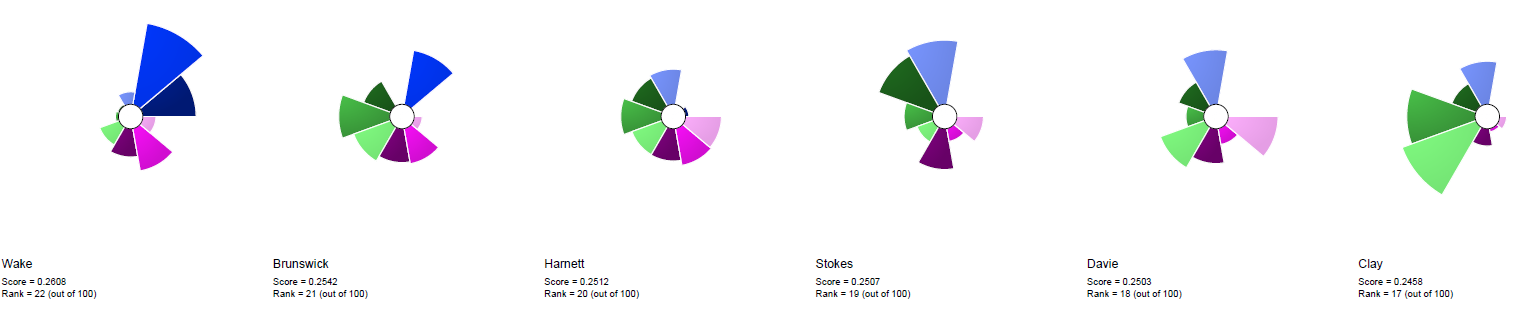  Brunswick  Rank = 21 |
| 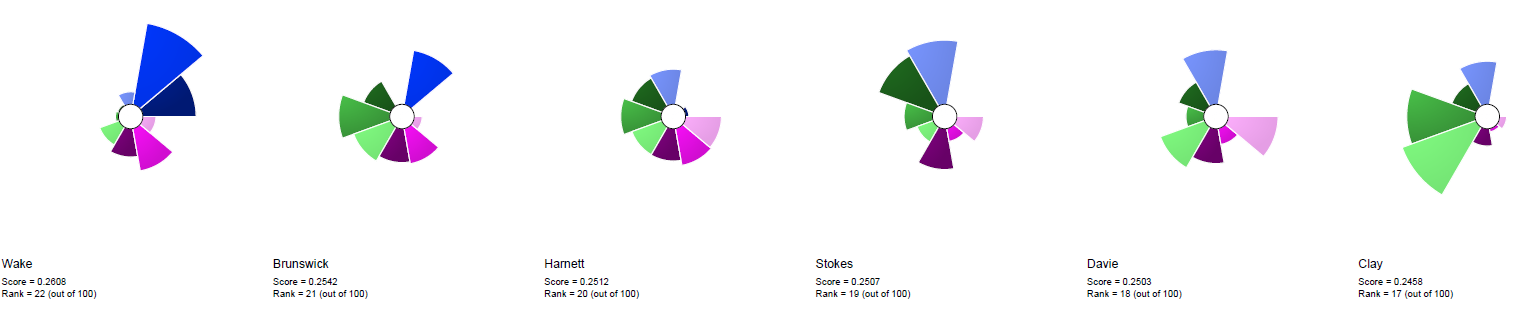  Harnett  Rank = 20 | 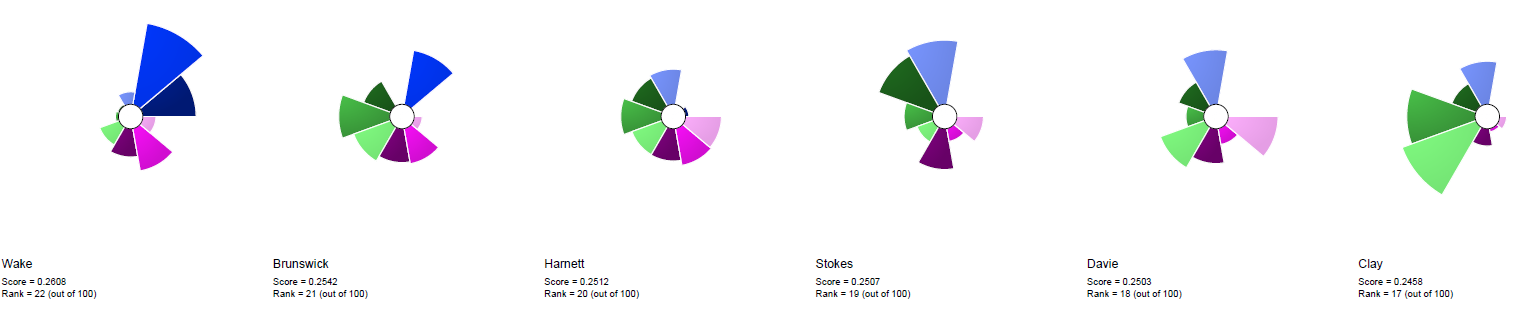  Stokes  Rank = 19 | 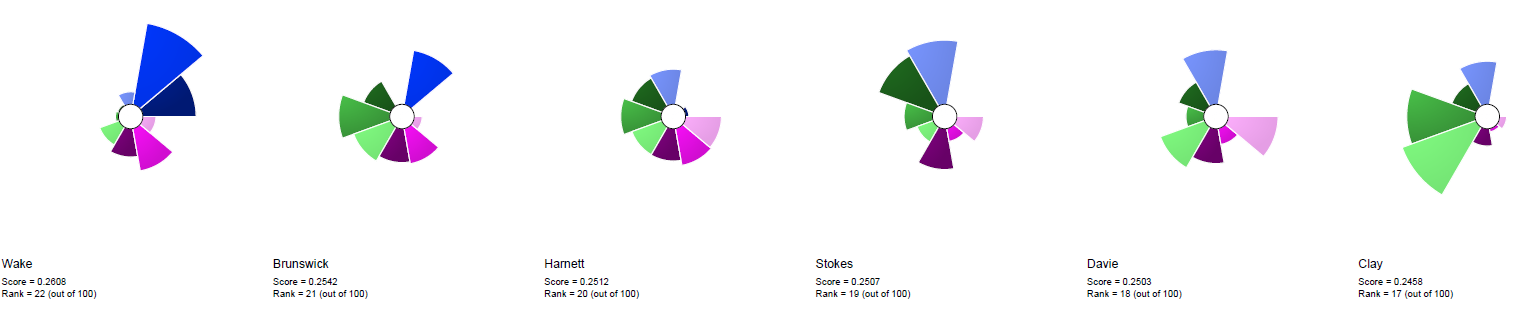  Davie  Rank = 18 | 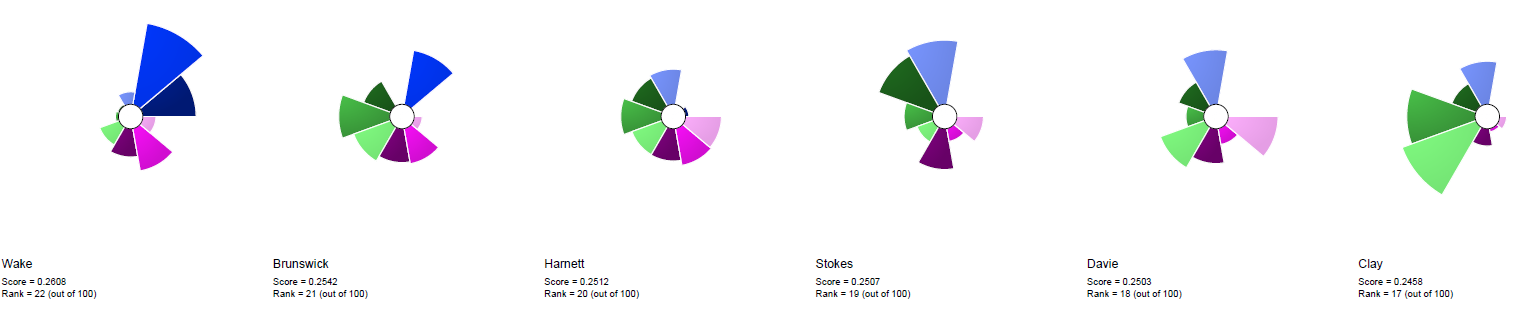  Clay  Rank = 17 | 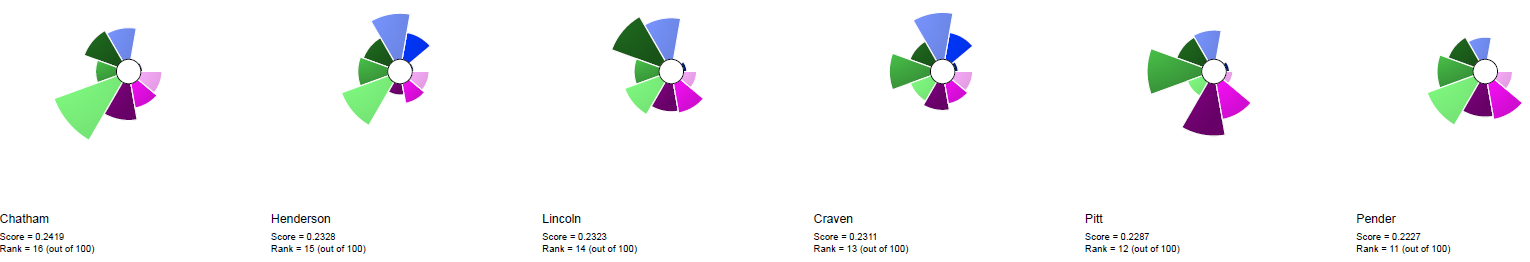  Chatham  Rank = 16 |
| 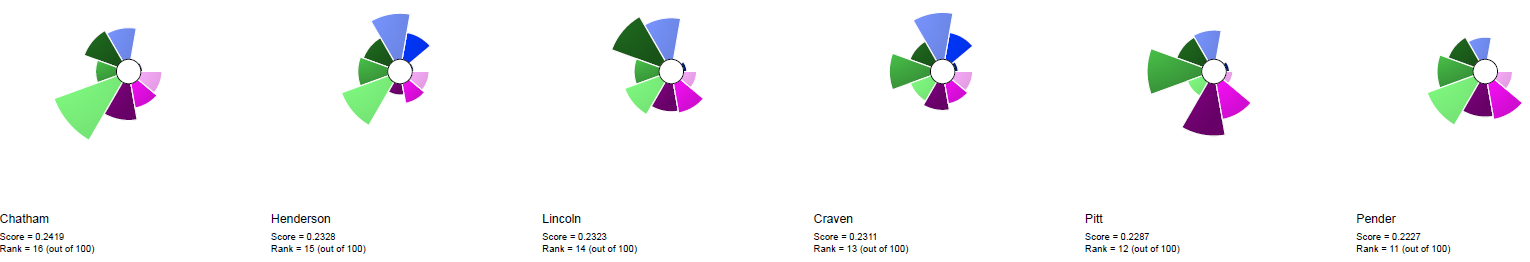  Henderson  Rank = 15 | 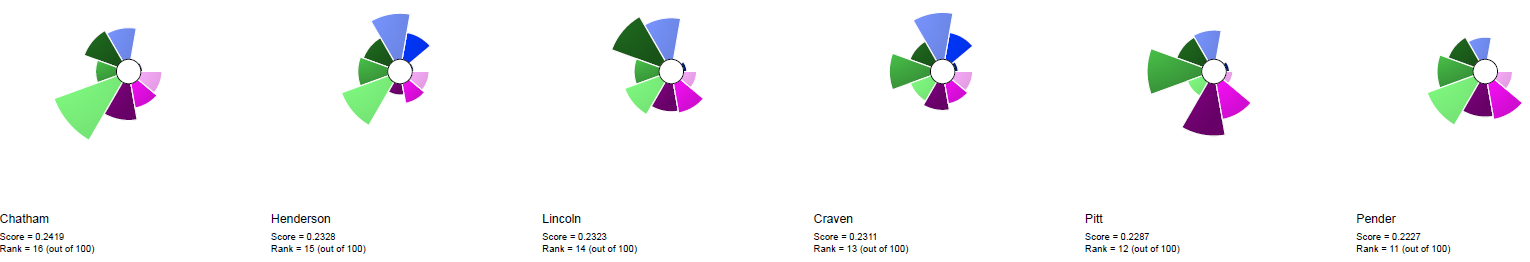  Lincoln  Rank = 14 | 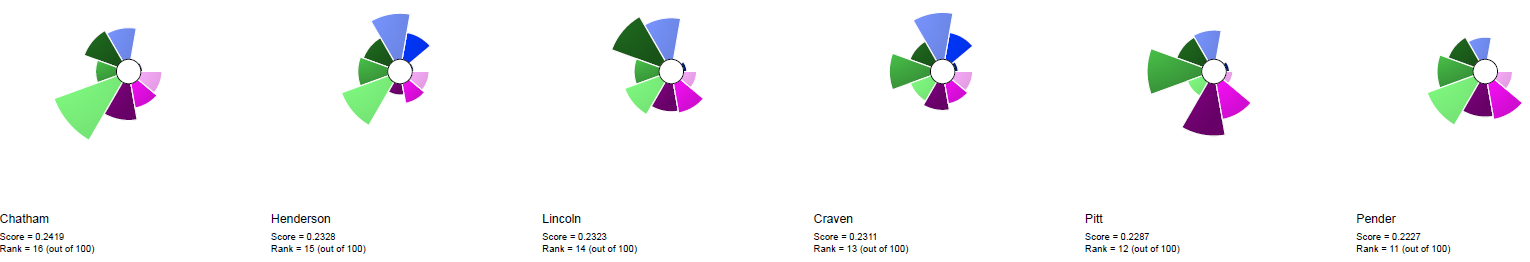  Craven  Rank = 13 | 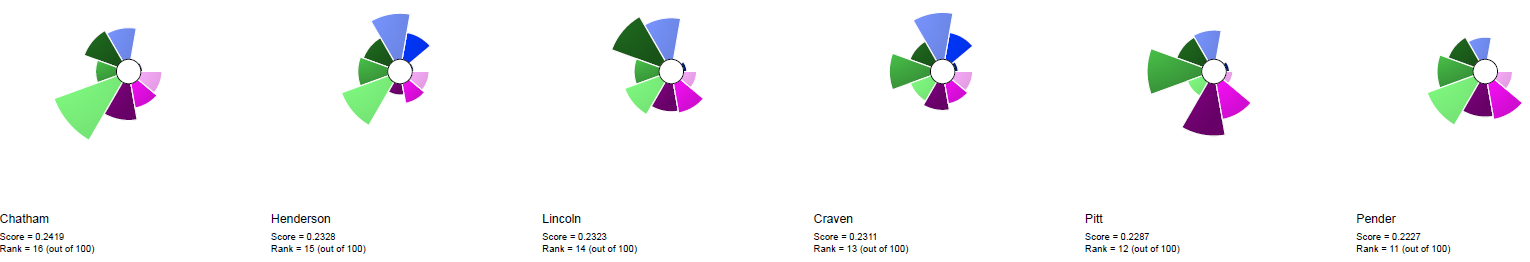  Pitt  Rank = 12 | 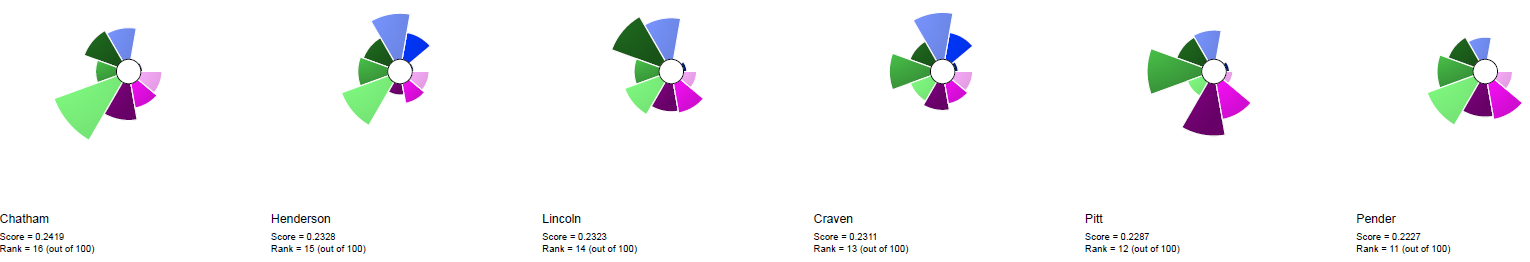  Pender  Rank = 11 |
| 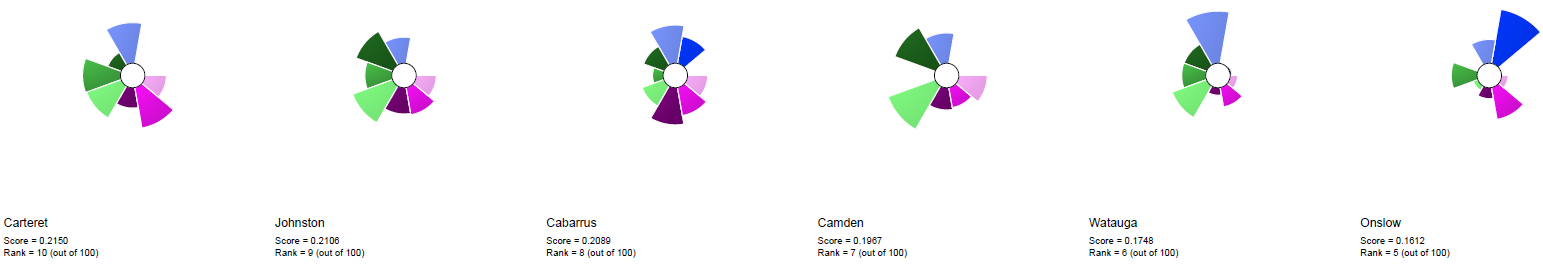  Carteret  Rank = 10 | 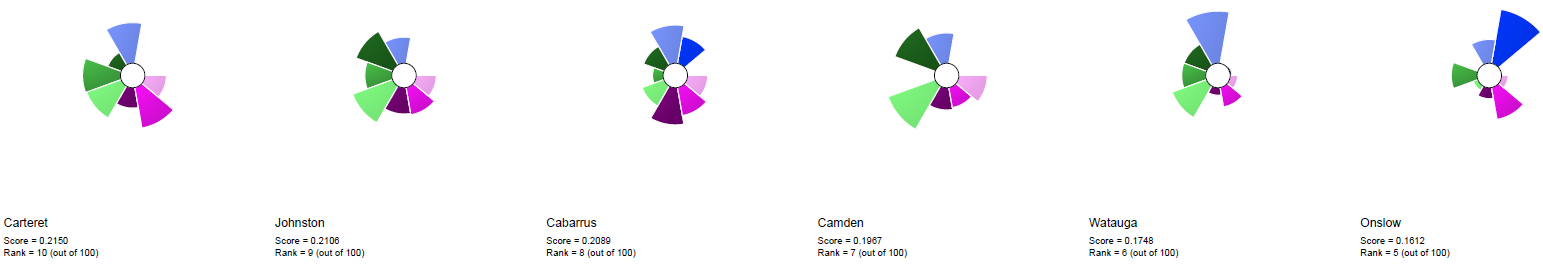  Johnston  Rank = 9 | 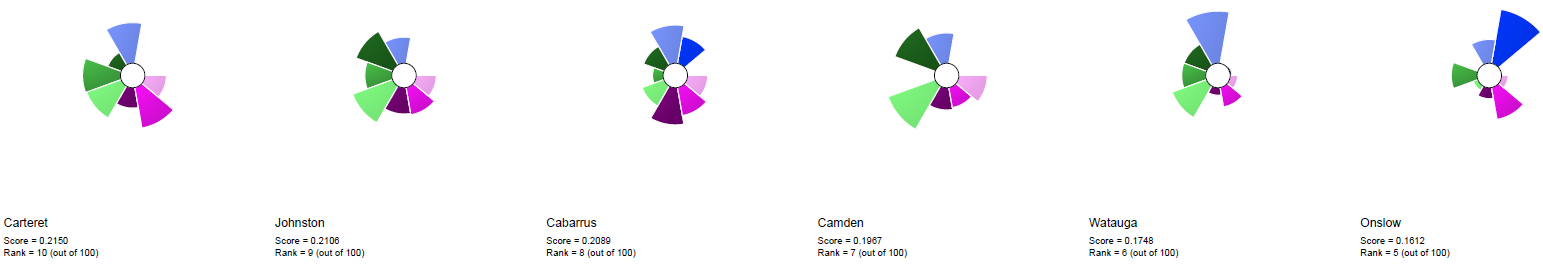  Cabarrus  Rank = 8 | 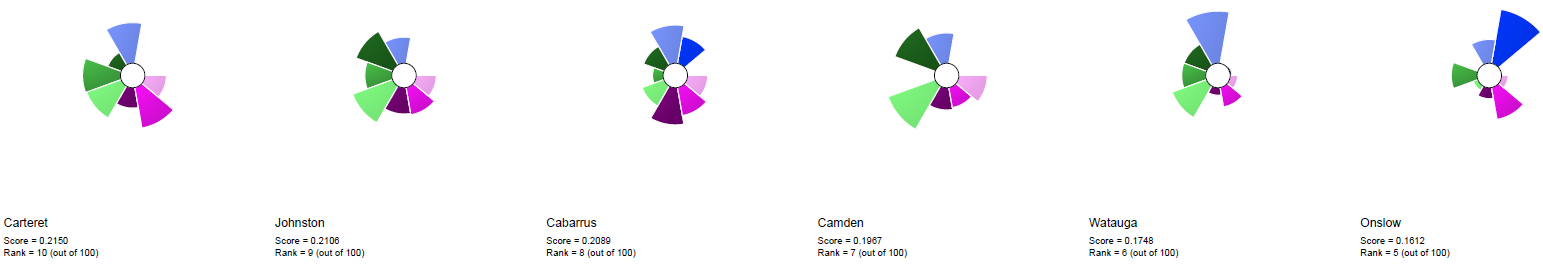  Camden  Rank = 7 | 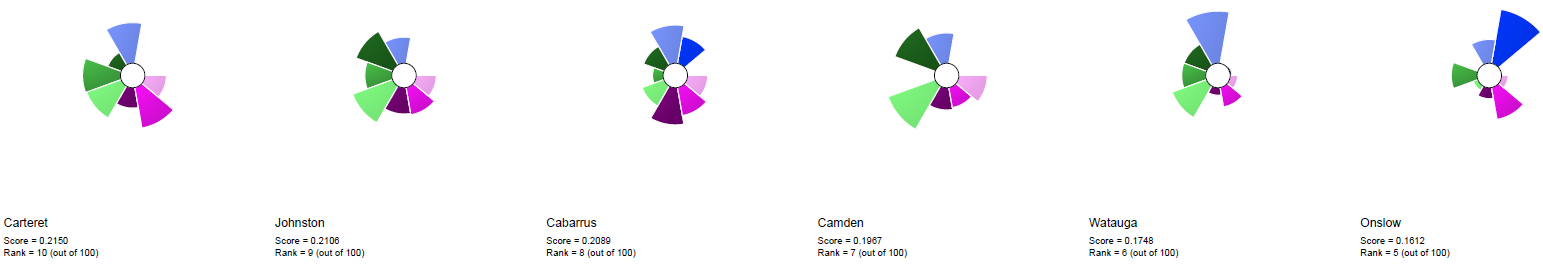  Watauga  Rank = 6 |
| 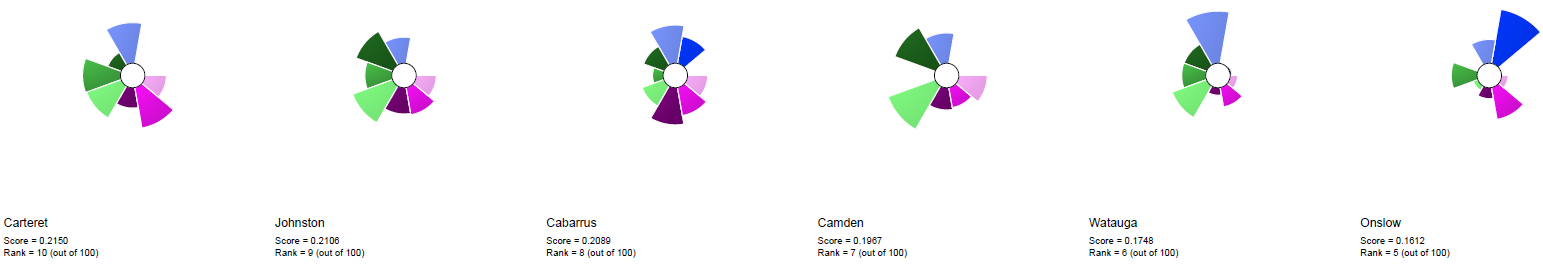  Onslow  Rank = 5 | 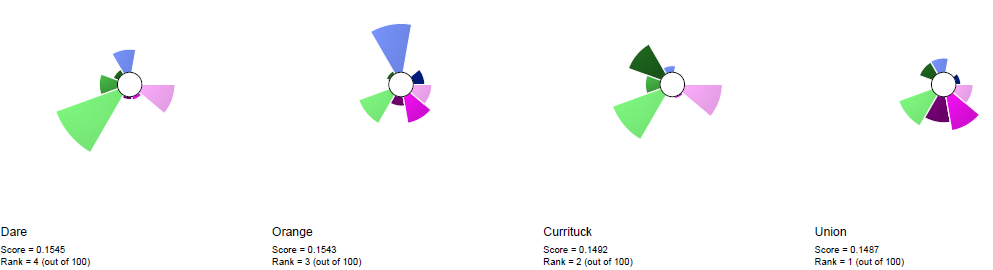  Dare  Rank = 4 | 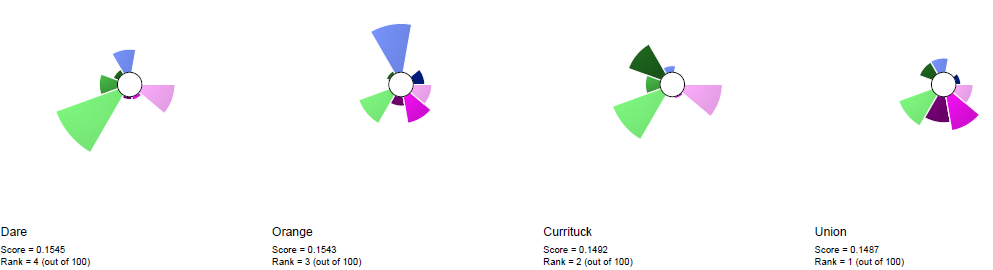  Orange  Rank = 3 | 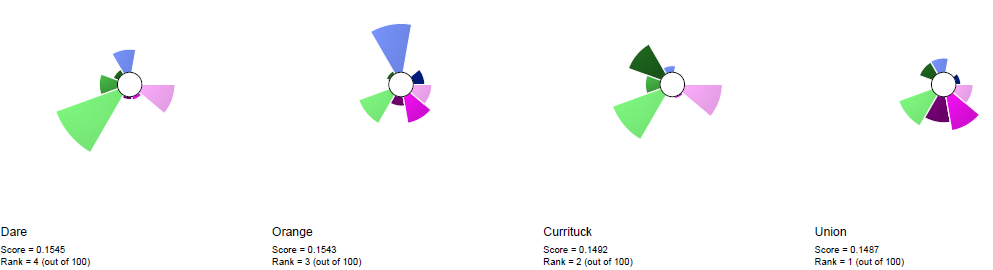  Currituck  Rank = 2 | Union  Rank = 1 |

*Figure S2. ToxPi charts for each North Carolina county ranked by vulnerability (vulnerability increases with rank number)*

*Figure S3: K-means Cluster Analysis Results. Used to determine the optimal number of groups of counties with similar vulnerability characteristics.*

*Figure S4. Hierarchical structure for 5 cluster groups of ToxPi county charts.*

*Figure S5. Map of Hierarchical Cluster Group 1.*

*This cluster includes counties with high percentages of homes built before 1979 (light blue), children under age 19 living below the poverty line (middle green), and children under age 15 that have been discharged from the hospital due to asthma (middle pink). These counties are more in the middle of NC counties based on percentage of the population under age 5.*

*Figure S6. Map of Hierarchical Cluster Group 2.*

*This cluster includes counties with a higher percentages of homes built before 1979 (light blue), children under age 19 living below the poverty line (middle green), babies born at a low birthweight (dark pink), and children under age 15 released from the hospital due to asthma (middle pink), though with less impact than in Group 1.*

*Figure S7. Map of Hierarchical Cluster Group 3.*

*This cluster of counties shows more impacts of industrial sources in the physical environment, Brownfield sites (dark blue) and Superfund sites (middle blue), and health outcomes.*

*Figure S8. Map of Hierarchical Cluster Group 4.*

*This cluster of counties does not show significant influences from any of the factors as seen in small ToxPi slices.*

*Figure S9. Map of Hierarchical Cluster Group 5.*

*This cluster of counties shows greater influences from homes built before 1979 (light blue), children under 19 living without insurance (light green), and with some areas having influence with head of household lacking a high school education (dark green).*
